# Spatiotemporal distributions, co-occurrence networks, and assembly mechanisms of the bacterial community in sediments of the Yangtze River: comprehensive insights into abundant and rare taxa

**DOI:** 10.3389/fmicb.2024.1444206

**Published:** 2024-12-11

**Authors:** Guohua Zhang, Shufeng Liu, Wenran Du, Yinghao Li, Zongzhi Wu, Tang Liu, Yichu Wang

**Affiliations:** ^1^Key Laboratory of Water and Sediment Sciences, Ministry of Education, College of Environmental Sciences and Engineering, Peking University, Beijing, China; ^2^College of Resources and Environmental Sciences, China Agricultural University, Beijing, China; ^3^School of Environment and Energy, Peking University Shenzhen Graduate School, Shenzhen, China; ^4^College of Chemistry and Environmental Engineering, Shenzhen University, Shenzhen, China; ^5^College of Water Sciences, Beijing Normal University, Beijing, China

**Keywords:** the Yangtze River, sedimentary bacteria, abundant and rare taxa, biogeography, co-occurrence network, assembling processes

## Abstract

Sediments are key reservoirs for rare bacterial biospheres that provide broad ecological services and resilience in riverine ecosystems. Compared with planktons, there is a lack of knowledge regarding the ecological differences between abundant and rare taxa in benthic bacteria along a large river. Here, we offer comprehensive insights into the spatiotemporal distributions, co-occurrence networks, and assembly processes of three divided categories namely always rare taxa (ART), conditionally rare taxa (CRT), and conditionally rare and abundant taxa (CRAT) in sediments covering a distance of 4,300 km in the Yangtze River. Our study demonstrated that ART/CRT contributed greatly to the higher Chao-1 index, Shannon-Wiener index, and phylogenetic alpha diversity of benthic bacteria in autumn than in spring. ART showed high overall beta diversity, and CRT/CRAT exhibited more significant distance-decay patterns than ART in both seasons, mainly corresponding to macroscopic landform types. CRT predominated the nonrandom co-occurrence network, with 97% of the keystone species mostly affiliated with Acidobacteriota flourishing in the lower-reach plain. Two selection processes had the greatest influences on the assembly of CRT (74.7–77.6%), whereas CRAT were driven primarily by dispersal limitation (74.9–86.8%) and ART were driven by heterogeneous selection (33.9–48.5%) and undominated stochasticity (32.7–36.5%). Natural factors such as river flow and channel slope exhibited more significant correlations with community variation than nutrients in all three groups, and total organic carbon mediated the balance among the distinct assembly processes of the ART and CRT in both seasons. Taken together, these results provide an improved ecological understanding of the discrepancy in biogeographic patterns between abundant and rare bacterial taxa in the sediments of Asia’s largest river.

## 1 Introduction

Owing to intricate interactions with various ecosystems through surface runoff and tributaries spanning continents, rivers play an essential role in global biogeochemical cycles and energy flows and are intrinsically linked to human activities and health (Aufdenkampe et al., 2011; Li K. et al., 2021; Liu et al., 2018, 2020). Within riverine systems, sediments function as both sources and sinks for various substances, with benthic bacteria occupying a vital position as primary drivers of metabolic activities (Gibbons et al., 2014). These microorganisms not only serve as crucial indicators of ecological stability (Roberto et al., 2018; Zhang et al., 2019) but also fundamentally contribute to essential biogeochemical processes such as the carbon, nitrogen, phosphorus, and sulfur cycles, as well as organic matter decomposition and mineralization (Huang et al., 2015; Yi et al., 2021). The sensitivity of benthic bacteria to subtle physical and chemical changes, coupled with their rapid growth rates, endows them with the capacity to exhibit diverse responses to environmental disturbances, thereby revealing community variations over time and space (Roberto et al., 2018; Yi et al., 2021). A recent study has indicated that the diversity of benthic bacteria in a world’s large river had a more substantial contribution than that of planktonic bacteria to the entire ecosystem (Liu et al., 2018). Consequently, delving into the spatiotemporal distribution and interaction patterns of benthic bacteria could greatly facilitate a deeper comprehension of river health and the operational dynamics of ecosystem services (Chen et al., 2019).

Comprehensively elucidating the structures and functions of microbial-driven ecosystems necessitates a nuanced analysis of the differences between abundant and rare taxa. Traditional microbial research methodologies have focused primarily on a few dominant taxa (abundant taxa), yet high-throughput sequencing technologies have revolutionized our understanding by revealing the extensive diversity of microorganisms with relatively low abundance, known as the “rare biosphere,” which play disproportionately significant roles within communities (Du et al., 2020; Jousset et al., 2017; Qin et al., 2022b; Yang et al., 2022). Abundant taxa, distinguished by their substantial biomass, occupy a broad range of ecological niches and contribute substantially to ecosystem functions (Jiao and Lu, 2020; Wan et al., 2021a; Yang et al., 2022). Conversely, rare taxa exhibit relatively high metabolic activity in specialized areas, thereby conferring unique and resilient ecological benefits (Lynch and Neufeld, 2015; Pester et al., 2010; Xue et al., 2018). Emerging evidence has suggested that abundant and rare taxa may exhibit distinct distribution patterns stemming from their specific assembly processes and adaptation strategies (Yi et al., 2022; Zhu et al., 2023). Thus, delving into the disparities between abundant and rare taxa presents a great opportunity to obtain a holistic perspective on how microorganisms drive and regulate ecosystem structure and function (Jiao and Lu, 2020; Wu et al., 2017; Yi et al., 2022). While extensive studies have been conducted on abundant and rare microbial communities in lakes (Bai et al., 2020; Zhang T. et al., 2022), reservoirs (Liu et al., 2015; Yan et al., 2020), soils (Gao et al., 2020; Jiao and Lu, 2020), and oceans (Li L. et al., 2021; Mo et al., 2018), there remains a significant shortage in research on the abundant and rare taxa in large natural rivers (Yi et al., 2022). Recent studies have shown that both abundant and rare microeukaryotes, as well as planktonic bacteria, share similar biogeographic patterns in subtropical rivers (Chen et al., 2019; Yi et al., 2022). Nevertheless, the specific roles of abundant and rare communities in shaping the geographic patterns of entire benthic bacterial communities in large rivers remain unclear.

The exploration of the intricate mechanisms that underlie microbial community assembly diversity is paramount to ecological understanding (Chen et al., 2019; Liu et al., 2018; Liu S. et al., 2022; Liu Y. et al., 2022; Yang et al., 2019). Two ecological process theories, niche and neutral theories, offer a comprehensive framework through which to comprehend the assembly structure of microbial communities within ecosystems. Niche theories (Bahram et al., 2016; Sloan et al., 2006) assert that the assembly and distribution of microbial communities are shaped by deterministic processes that are mediated by multiple biotic and abiotic factors. In contrast, neutral theories contend that stochastic processes, characterized by random fluctuations such as birth, death, migration, speciation, and dispersal limitation, play pivotal roles in shaping the establishment of microbial communities (Roguet et al., 2015). Discriminative methods based on null models have been widely utilized to assess the impacts of deterministic and stochastic processes (Stegen et al., 2012). Research has shown that the relative importance of these processes is significantly influenced by complex factors, including habitat type and physicochemical conditions (Jiao et al., 2020; Liu S. et al., 2022; Liu et al., 2023). Moreover, the patterns of microbial interactions and coexistence mechanisms have been extensively studied through analyses of non-randomness, topological characteristics, and keystone species in networks, providing a deeper understanding of the structure and dynamics of microbial communities (Liu et al., 2023; Ma et al., 2020). Recent studies have underscored the importance of biological interactions in influencing community assembly and ecological function, as evidenced by co-occurrence network analyses across diverse habitats (Hu et al., 2017; Liu et al., 2023; Ma et al., 2016; Qin et al., 2022a; Wang et al., 2020). While investigations on the assembly and coexistence patterns of microbial communities in river ecosystems have been conducted, they focused predominantly on planktonic bacteria (Wang et al., 2020; Wu et al., 2023; Yi et al., 2022; Zhang T. et al., 2022; Zhu et al., 2023). There remains a notable knowledge gap regarding the assembly processes and coexistence strategies of riverine benthic bacteria, particularly when distinguishing between abundant and rare taxa.

Focusing on the Yangtze River, which is the longest in Asia and the third-longest globally (Liu et al., 2018), our present study aimed to explore the spatial distribution, coexistence patterns, and assembly mechanisms of abundant and rare benthic bacterial taxa using 16S rRNA high-throughput sequencing strategy. In both spring and autumn, we positioned 24 sampling sties from Shigu to Xuliujing in the mainstream, which captures a variety of landforms from mountainous to plain regions. Our primary research objectives were to (i) elucidate the biogeographical and coexistence dynamics of abundant and rare benthic bacteria; (ii) uncover the underlying assembly mechanisms that govern the structure of abundant and rare sedimentary bacterial communities; and (iii) reveal the impacts of various environmental factors on the assembly dynamics of abundant and rare communities within the Yangtze River ecosystem.

## 2 Materials and methods

### 2.1 Sample collection and environmental factors

The Yangtze River originates from the Qinghai-Tibet Plateau and traverses 11 provinces in China. It has a total curvilinear length of approximately 6,300 kilometers and a drainage basin of around 1.8 million km^2^ (Liu et al., 2018). To investigate the spatiotemporal distribution and assembly mechanisms of sedimentary bacterial taxa, sediment sampling was conducted at 24 mainstream national hydrological stations along the Yangtze River in March 2014 (Spring) and October 2014 (Autumn) (Supplementary Figure S1). The geographical areas of the sampling points were categorized into five types: mountain, hill, basin, mountain-hill, and plain regions (Supplementary Table S1). No extreme weather events were observed during the sampling period, while collection at a few monitoring sites was hindered by rugged terrain and swift flow conditions. Additionally, replicated sampling (1–4 samples) was performed at locations with heterogeneous sediments. Sediment samples were collected from the surface layer (0–5 cm) of the river bed, sealed in 50 mL sterilized polypropylene tubes and transported to a nearby laboratory on dry ice for preservation at −80°C until DNA extraction.

Total organic carbon (TOC) and total nitrogen (TN) were quantified using a TOC/TN-VCPH analyzer (Shimadzu, Kyoto, Japan), and total phosphorus (TP) was determined via spectrophotometric methods in accordance with standard protocols (Liu et al., 2013). Water temperature (WT) was measured using a multi-parameter online analyzer (Shanghai San-Xin Instrumentation). Additional environmental parameters, including pH, ammonium nitrogen (NH_3_-N), and nitrate nitrogen (NO_3_-N), were measured according to the procedures outlined by Zhu et al. (2011). River flow was typically recorded using flow meters, and information on the channel slope of the Yangtze River was sourced from Chen et al. (2001). The spatial factors (latitude, longitude, and altitude) at each sampling site were recorded with a handheld GPS device (Magellan, United States).

### 2.2 PCR amplification and sequencing

Genomic DNA was extracted using the FastDNA^®^ SPIN Kit for Soil (MP Biomedicals, United States) by following the manufacturer’s instructions. Subsequently, the duplicate DNA extracts were combined and PCR was conducted to amplify the V4-V5 region of the 16S rRNA gene in each sample using primers 515F (5′-GTGCCAGCMGCCGCGG-3′) and 907R (5′-CCGT CAATTCMTTTRAGTTT-3′) (Shan et al., 2015). The reaction protocol included initial denaturation at 95°C for 2 min, followed by 25 cycles of denaturation at 95°C for 30 s, annealing at 55°C for 30 s, and elongation at 72°C for 30 s, with a final extension at 72°C for 5 min. PCRs were performed in triplicate in a 20 μL reaction mixture containing 4 μL of 5 × FastPfuBuffer, 2 μL of 2.5 mM dNTPs, 0.8 μL of each primer (5 μM), 0.4 μL of FastPfu polymerase, and 10 ng of template DNA. The PCR products were visualized on 2% agarose gels, purified using the AxyPrep DNA Gel Extraction Kit (Axygen Biosciences, Union City, CA, United States), quantified using QuantiFluor^™^-ST (Promega, United States) and sequenced on the Illumina MiSeq platform for paired-end sequencing with 2 × 250 bp strategy.

### 2.3 Bioinformatic analysis

The 16S rRNA sequencing results were subjected to a series of quality control and filtration procedures utilizing QIIME2 (Bolyen et al., 2019). Paired-end reads were merged and quality filtered using VSEARCH (Rognes et al., 2016). Subsequently, denoising and generation of amplicon sequence variants (ASVs) were then performed with the Deblur plugin (Amir et al., 2017). The obtained ASVs were taxonomically classified using the Silva 16S rRNA database (Quast et al., 2013) via Classify-sklearn (Bokulich et al., 2018), excluding ASVs identified as mitochondria or chloroplasts. A phylogenetic tree was constructed using FastTree2 (Price et al., 2020). To equalize sequencing depth, each dataset was rarefied to the lowest number of clean reads across all the samples.

### 2.4 Definitions of abundant and rare taxa

Following the classification criteria proposed for abundant and rare taxa based on the previous analysis (Chen et al., 2019), a relative abundance threshold of 0.01% was defined for rare taxa, whereas a threshold of 1% was set for abundant taxa. To delve deeper into the composition and dynamics of relatively rare taxonomic groups within the benthic bacterial communities, six classes of ASVs were categorized: always abundant taxa (AAT) were identified as ASVs with an abundance ≥1% across all the samples; conditionally abundant taxa (CAT) were identified as ASVs with an abundance ≥0.01% in all the samples and ≥1% in some samples; conditionally rare and abundant taxa (CRAT) were identified as ASVs with an abundance ranging from rare (<0.01%) to abundant (≥1%); moderate taxa (MT) were identified as ASVs with an abundance ranging from 0.01 to 1% across all the samples; conditionally rare taxa (CRT) were defined as ASVs that consistently maintained an abundance less than 1% in all the samples and below 0.01% in some samples; always rare taxa (ART) were identified as ASVs with an abundance <0.01% in all the samples. In the Yangtze River, we identified only three taxonomic groups among all the ASVs: ART, CRT and CRAT. CRAT were classified as abundant taxa, and both CRT and ART were categorized as rare taxa, facilitating a more detailed comparative exploration.

### 2.5 Statistical analysis

#### 2.5.1 Alpha and beta diversity analysis

Alpha diversity indices (Chao1, Shannon-Wiener, and phylogenetic diversity) were calculated for the bacterial communities in the spring and autumn sediment samples using the *vegan* package in R-4.0.0. Concurrently, Wilcoxon rank-sum tests were utilized to assess the significance of the differences of: (1) all the alpha diversity indices between spring and autumn; and (2) the single ASV abundance, the ASV number, and all-ASV abundance among the ART, CRT, and CRAT groups, or between two seasons. Beta diversity analysis was conducted to evaluate the community dissimilarities of ART, CRT, and CRAT by calculating the Bray–Curtis and unweighted UniFrac distances with the *vegan* package in R-4.0.0. To assess the significance of seasonal and spatial variations, analysis of similarities (ANOSIM) was performed using the *vegan* package in R-4.0.0.

#### 2.5.2 Distance-decay patterns

The distance-decay patterns for community similarity of ART, CRT, and CRAT were evaluated based on the geographical distances from each sampling site to the river mouth (Liu et al., 2020), which was calculated using ArcGIS v10.2 software. The rate of distance decay was determined by the slopes obtained from linear least squares regression analyses, which correlated geographical distances with community similarity metrics using the Bray–Curtis and unweighted UniFrac distance matrices.

#### 2.5.3 Co-occurrence network analysis

Network analysis was performed using the Hmisc and igraph packages in R-4.0.0 and visualized using Gephi v0.9.2^1^ to elucidate the co-occurrence patterns of benthic bacterial communities. Spearman’s correlation coefficients were calculated, excluding ASVs with sample detection rates less than 20%. Correlations with a coefficient *r* > 0.8 and a *p*-value <0.01 were considered robust and significant. A series of node-level topological parameters such as node degree, betweenness centrality, and closeness centrality were calculated, and Wilcoxon rank-sum tests were used to examine the difference significance of these parameters between the major phyla. Additionally, 10,000 Erdős–Rényi random network possessing an equivalent number of nodes and edges as the real network was generated to compare actual connections against a baseline of randomness (Hu et al., 2017). Keystone species, identified as nodes with high connectivity (high degree), play an integral role in the structure and function of microbial communities (Mohapatra et al., 2022). Following the criteria of Ma et al. (2016), the keystone species were identified with a node degree >100 and betweenness centrality <5,000. The observed (O) value represents actual connections between nodes, and the random (R) value suggests expected connections under random conditions (Hu et al., 2017). The O/R ratio serves as an index to measure non-randomness in co-occurrence patterns, and the O/R ratio >1 indicates that the observed co-existence incidence of two taxa is higher than random (Liu et al., 2023).

#### 2.5.4 Community assembly mechanism analysis

The beta nearest taxon index (βNTI) metric (Stegen et al., 2013) could serve as a valuable indicator for assessing the degree to which deterministic or stochastic factors influence changes between various communities and has been extensively applied in recent studies (Chen et al., 2019; Liu et al., 2023; Yi et al., 2022). |βNTI| >2 suggests that community turnover is primarily governed by selection, and βNTI >2 and <−2 indicate heterogeneous selection and homogeneous selection, respectively. |βNTI| <2 suggests that the community turnover is driven primarily by stochastic processes. Subsequently, the Raup–Crick matrix (RCbray), derived from the standard Bray–Curtis matrix of community composition, is calculated to further decouple the contribution of stochastic processes. Within this framework, |βNTI| <2 and RCbray >0.95 could be interpreted as dispersal limitation, |βNTI| <2 and RCbray <−0.95 could be identified as homogenizing dispersal, and |βNTI| <2 and |RCbray| <0.95 indicate that no single process dominates stochastic assembly.

#### 2.5.5 Impacts of environmental factors on community structure and assembly mechanisms

Mantel tests between environmental factors and the variations of three bacterial sub-communities based on Bray–Curtis distances and βNTI matrices were conducted to examine the correlation using the *vagan* package in R-4.0.0. Partial Mantel tests, with geographical distance as the control variable, were further used to investigate the relationships between various environmental factors and community assembly mechanisms. Variance partitioning analysis (VPA), which is based on redundancy analysis (RDA), was performed to elucidate the influence of various environmental factors on the structure of sedimentary bacterial communities using the *vegan* package in R-4.0.0.

## 3 Results

### 3.1 Alpha diversity and community composition of sedimentary bacteria between spring and autumn

**Figure 1:**
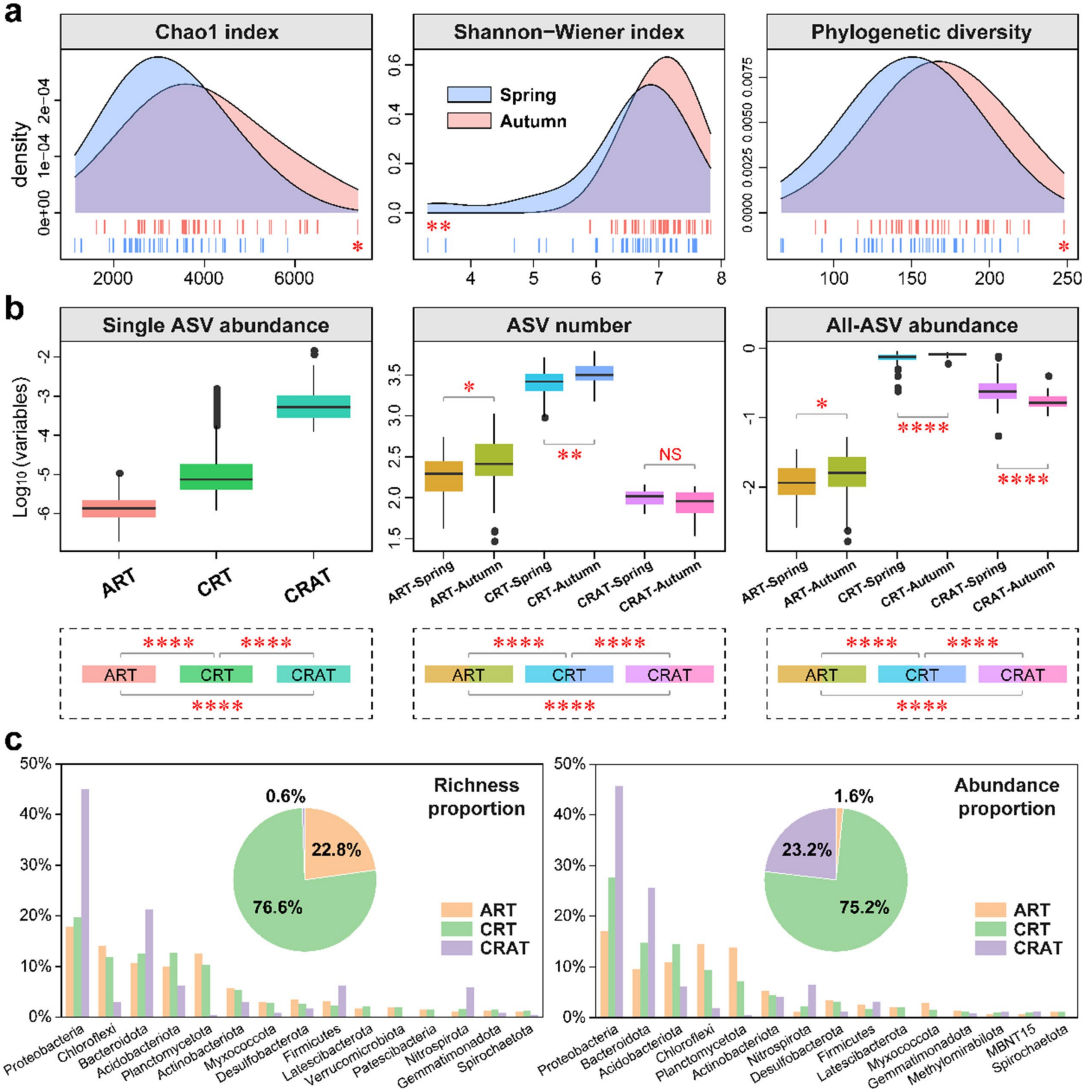
Alpha diversity and community composition of benthic bacterial ART, CRT and CRAT in the spring and autumn seasons. **(A)** Density plots showing the alpha diversity (Chao1, Shannon-Wiener and phylogenetic diversity indices) of the bacterial communities in the spring and autumn samples, and statistical analysis was performed via Wilcoxon rank-sum tests (^**^p < 0.01 and ^*^p < 0.05). **(B)** Boxplots showing the relative abundance of individual ASV, the number of ASVs, and the community relative abundance of ART, CRT and CRAT in the spring and autumn samples. Statistical analysis was performed via Wilcoxon rank-sum tests (^****^p < 0.0001, ^**^p < 0.01, and ^*^p < 0.05). **(C)** Pie charts representing the richness and abundance proportions of ART, CRT, and CRAT, and bar charts showing the richness and abundance proportions of the three subcommunities in the top 15 phyla.

A total of 42,304 ASVs were identified by high-throughput sequencing of sediment samples from 24 sites along the mainstream of the Yangtze River (Supplementary Figure S1 and Supplementary Table S1). Notably, the Good’s coverage values for individual samples ranged from 87% to 97% (Supplementary Table S2), indicating that the sequencing depths were sufficient to cover the majority of bacterial species. Furthermore, significant seasonal variations (*p* < 0.05) were observed in the richness index (Chao1) and diversity indices (Shannon-Wiener and phylogenetic diversity), with autumn exhibiting higher values than spring (Figure 1A), indicating that seasonal changes played a pivotal role in shaping the alpha diversity of sedimentary communities in the Yangtze River.

The ART, CRT, and CRAT groups were defined and categorized on the basis of the relative abundance differences of individual ASVs within samples (Figure 1B). ART contained 9,647 ASVs (22.8%), CRT were comprised of 32,417 ASVs (76.6%), and CRAT consisted of only 240 ASVs (0.6%) (Figure 1C). Notably, while CRAT ASVs were significantly (*p* < 0.0001) rarer than ART and CRT in terms of the total number, they exhibited higher individual ASV abundances (*p* < 0.0001) (Figure 1B). However, for the total number and abundance, CRT exhibited the highest, followed by CRAT and ART. Seasonal variations were also evident, with ART and CRT showing significantly higher numbers and abundances in autumn than in spring (*p* < 0.0001) (Figure 1B). In contrast, CRAT exhibited a significant seasonal difference only in terms of overall abundance, with an opposite trend to ART and CRT (Figure 1B).

Regarding the total richness and abundance proportions, CRT (richness: 76.6%; abundance: 75.2%) contributed much more than ART (richness: 0.6%; abundance: 23.2%) and CRAT (richness: 22.8%; abundance: 1.6%) (Figure 1C). At phylum level, Proteobacteria, Chloroflexi, Bacteroidota, Acidobacteriota, Planctomycetota and Actinobacteriota emerged as the most prevalent phyla in the bacterial community of the Yangtze River sediment. Among these phyla, Proteobacteria and Bacteroidota dominated the richness and abundance of CRAT, those of CRT were dominated by Proteobacteria, Bacteroidota, and Acidobacteriota, and those of ART were dominated by Proteobacteria, Chloroflexi, and Planctomycetota (Figure 1C). Further taxonomic analysis at class level revealed that the dominant classes included Gammaproteobacteria, Anaerolineae, Bacteroidia, Alphaproteobacteria, Vicinamibacteria, and Planctomycetes (Supplementary Figure S2). Among these classes, Gammaproteobacteria and Bacteroidia dominated the richness and abundances of CRAT and CRT, and Gammaproteobacteria, Anaerolineae, and Planctomycetes dominated the richness and abundance of ART (Supplementary Figure S2).

### 3.2 Spatial patterns of the sedimentary bacterial community

**Figure 2:**
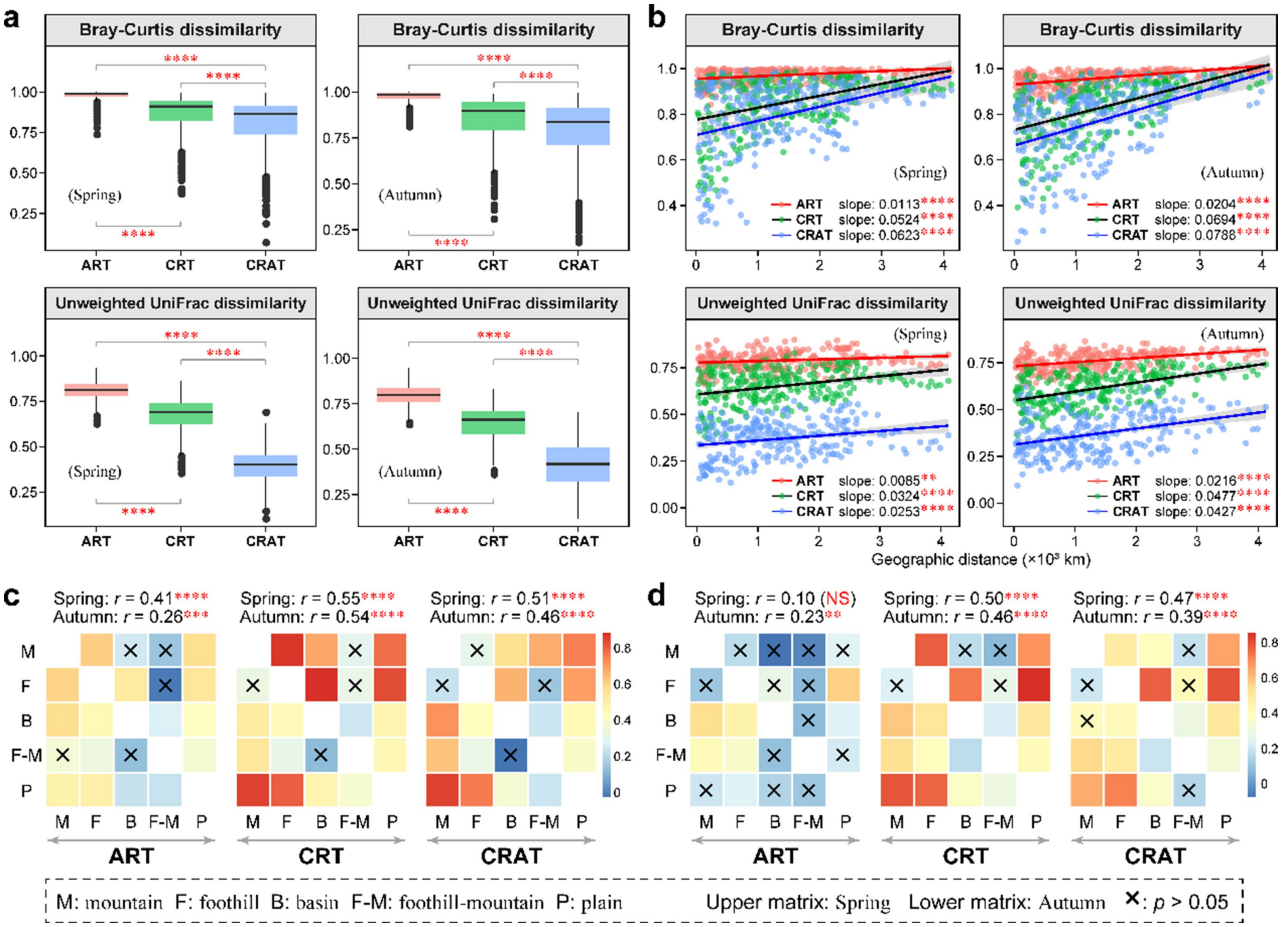
Spatial turnover of the benthic bacterial ART, CRT, and CRAT in spring and autumn. **(A)** Boxplots showing the beta diversity of the ART, CRT, and CRAT in spring and autumn, and statistical analysis was performed using Wilcoxon rank-sum tests (^****^p < 0.0001). **(B)** Distance-decay patterns of the ART, CRT, and CRAT in spring and autumn examined using a permutation test (9,999 permutations) based on the Bray–Curtis and unweighted UniFrac distances (^****^p < 0.0001 and ^**^p < 0.01). **(C,D)** Heatmaps showing the pairwise ANOSIM *r*-values between any two landform types for ART, CRT, and CRAT based on the Bray–Curtis distance **(C)** and Unweighted UniFrac distance **(D)**. The upper triangular matrix represents spring data and the lower triangular matrix shows autumn data, with the global ANOSIM *r-* and *p*-values among the five landform types shown on the top of the heatmaps (^****^p < 0.0001, ^***^p < 0.001, ^**^p < 0.01, and ^*^p < 0.05).

The beta diversity of ART, CRT and CRAT across the sediment samples was analyzed. NMDS analysis based on the Bray–Curtis and unweighted UniFrac distances revealed significant seasonal variations (ANOSIM *r* = 0.10–0.16, *p* = 0.001) in the ART, CRT, CRAT, and total bacterial communities (Supplementary Figure S3). Beta diversity analysis reinforced the distinctiveness among the ART, CRT, and CRAT communities, however, the consistent diversity distribution trends across both seasons were generally maintained (Figure 2). During both the high-flow (autumn) and low-flow (spring) periods, ART and CRT exhibited higher community Bray–Curtis and unweighted UniFrac dissimilarities than CRAT, suggesting greater beta diversity within the rare taxa (ART and CRT) than among the abundant taxa (CRAT) (Figure 2A). Significant distance-decay patterns of ART, CRT, and CRAT were observed (*p* < 0.01), as their community Bray–Curtis and unweighted UniFrac similarities decreased accompanied with the increasing geographical distance in both spring and autumn (Figure 2B). Notably, the linear regression slopes for CRT (spring: 0.0524/0.0324, autumn: 0.0694/0.0477) and CRAT (spring: 0.0623/0.0253, autumn: 0.0788/0.0427) were steeper than those for ART (spring: 0.0113/0.0085, autumn: 0.0204/0.0216), indicating a more pronounced decline in similarity with increasing distance for these groups. Moreover, the steeper slopes observed in autumn than in spring suggested that these taxa exhibited greater spatial variations in autumn.

To delve deeper into the spatial patterns of sedimentary bacterial communities, we categorized the samples based on their geomorphological characteristics, classifying them into five landforms: mountain, foothill, basin, foothill-mountain and plain. By utilizing both the Bray–Curtis distance and unweighted UniFrac distance, ANOSIM revealed significant differences (*p* < 0.01) in sediment community composition among these landforms during both seasons (except ART in spring) (Figures 2C,D). By comparing the global ANOSIM *r*-values, we found that CRT and CRAT were more significantly affected by landform types than ART, suggesting greater spatial variations in communities. Further pairwise ANOSIM demonstrated strong community differences of CRT and CRAT between the mountain or foothill and plain regions.

### 3.3 Co-occurrence patterns for the sedimentary community

**Figure 3:**
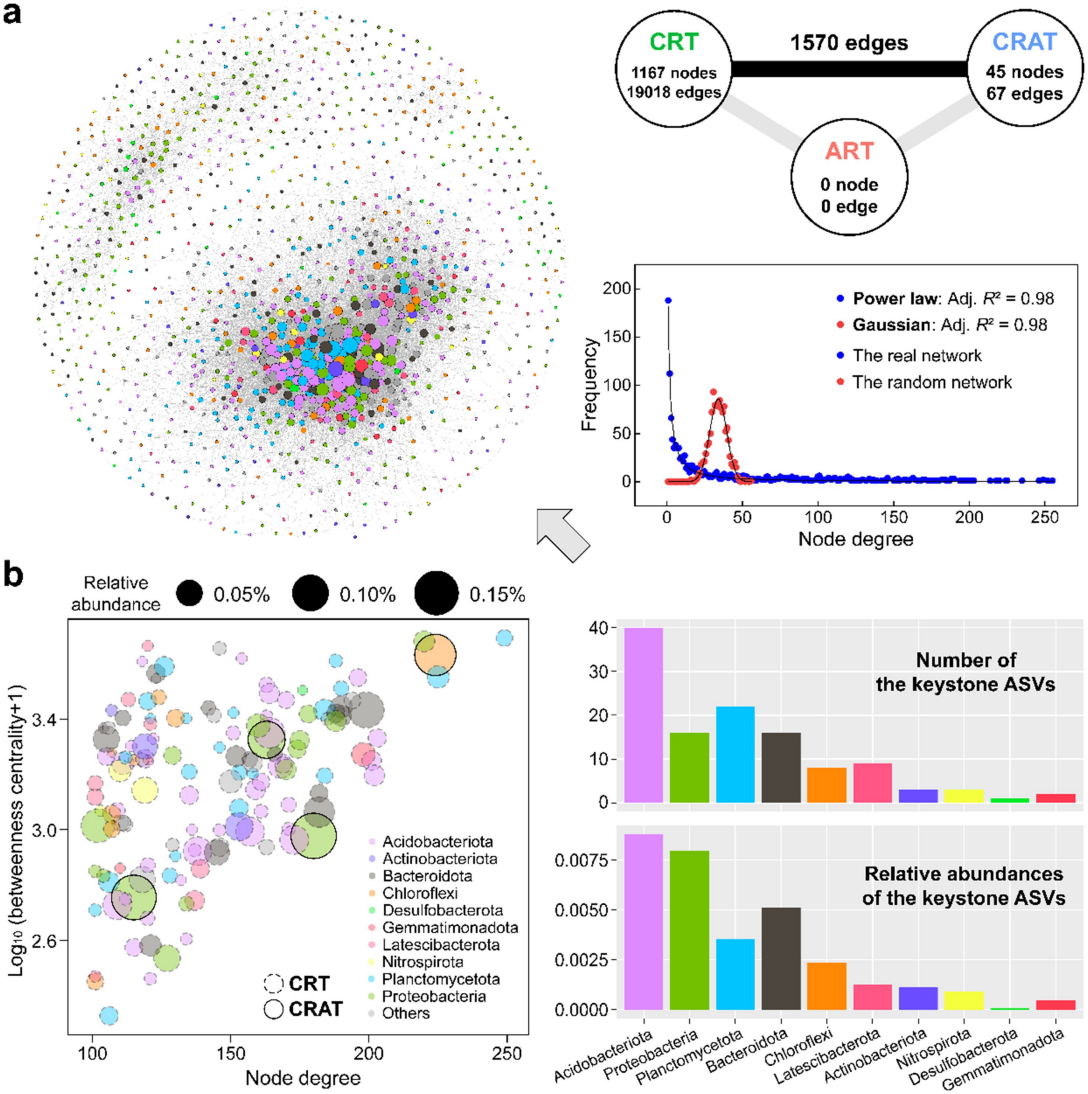
The coexistence network pattern of the sedimentary bacterial communities in the Yangtze River. **(A)** Co-occurrence network constructed based on the Spearman correlation between ASVs in sediment. Nodes in the network are colored by different phyla. Connections in the network indicate strong (Spearman’s *r* > 0.8) and significant (*p* < 0.01) correlations. The size of each node is proportional to the degree. On the right side are the node degree distributions of the benthic bacterial network (blue) and 10,000 Erdős–Rényi random network (red). The solid black lines represent the power-law and Gaussian best fits of the degree distribution for the real network and an Erdős–Rényi random network of equal size (^****^p < 0.0001). **(B)** Identification of the keystone ASVs in the network using the criteria of node degree >100 and betweenness centrality <5,000 (Ma et al., 2016).

Network analysis was conducted to uncover the co-occurrence patterns within the sedimentary communities of the Yangtze River (Figure 3). The resulting network comprised of 1,212 nodes and 20,655 edges, with 1,167 nodes belonging to CRT and just 45 nodes belonging to CRAT (Figure 3A). The node degree distribution of the real network obeyed a scale-free power law pattern (adj. *R*^2^ = 0.98), which is different from the Gaussian distribution for an Erdős–Rényi random network of the similar size (adj. *R*^2^ = 0.98), thereby indicating a non-random network structure for the sedimentary bacterial community in the Yangtze River.

Within the co-occurrence network, 136 keystone species (high-degree nodes) were identified, predominantly belonging to the phyla Acidobacteriota, Proteobacteria, Planctomycetota, Bacteroidota, and Chloroflexi (Figure 3B). These phyla exhibited intricate interrelations as well as significant advantages in terms of both keystone presence and relative abundance (Figure 3B and Supplementary Table S3). Among the keystone species, all nodes affiliated with Acidobacteriota, Planctomycetota, and Bacteroidota were classified as CRT, whereas three high-abundance nodes from Proteobacteria and one from Chloroflexi belonged to the CRAT. Acidobacteriota ASVs dominated both the number and relative abundance of keystone species in the network, and they mostly flourished in the lower-reach plain region.

At the phylum level, the top five phyla of the sedimentary bacterial community displayed significant differences (*p* < 0.05) in node degree and closeness centrality among most of them (Supplementary Figure S4). Both Acidobacteriota and Planctomycetota ASVs presented higher node degree and closeness centrality than the other three phyla.

Furthermore, the analysis of the O/R ratios among the different groups underscored their co-existence tendencies (Supplementary Table S4). ASVs within Planctomycetota and Acidobacteriota rather than other major phyla tended to co-exist more often than expected as revealed by relatively high O% (3.4–7.1%) and O/R ratios (1.7–2.9). Notably, there was also a relatively high incidence of inter-phylum co-existence than expected from the random associations between Acidobacteriota and many other phyla (O/R ratios up to 2.5) including a few dominant phyla, highlighting the central role of Acidobacteriota in shaping the nonrandom co-occurrence pattern within sedimentary bacterial community.

### 3.4 Community assembly mechanisms of sedimentary bacteria

**Figure 4:**
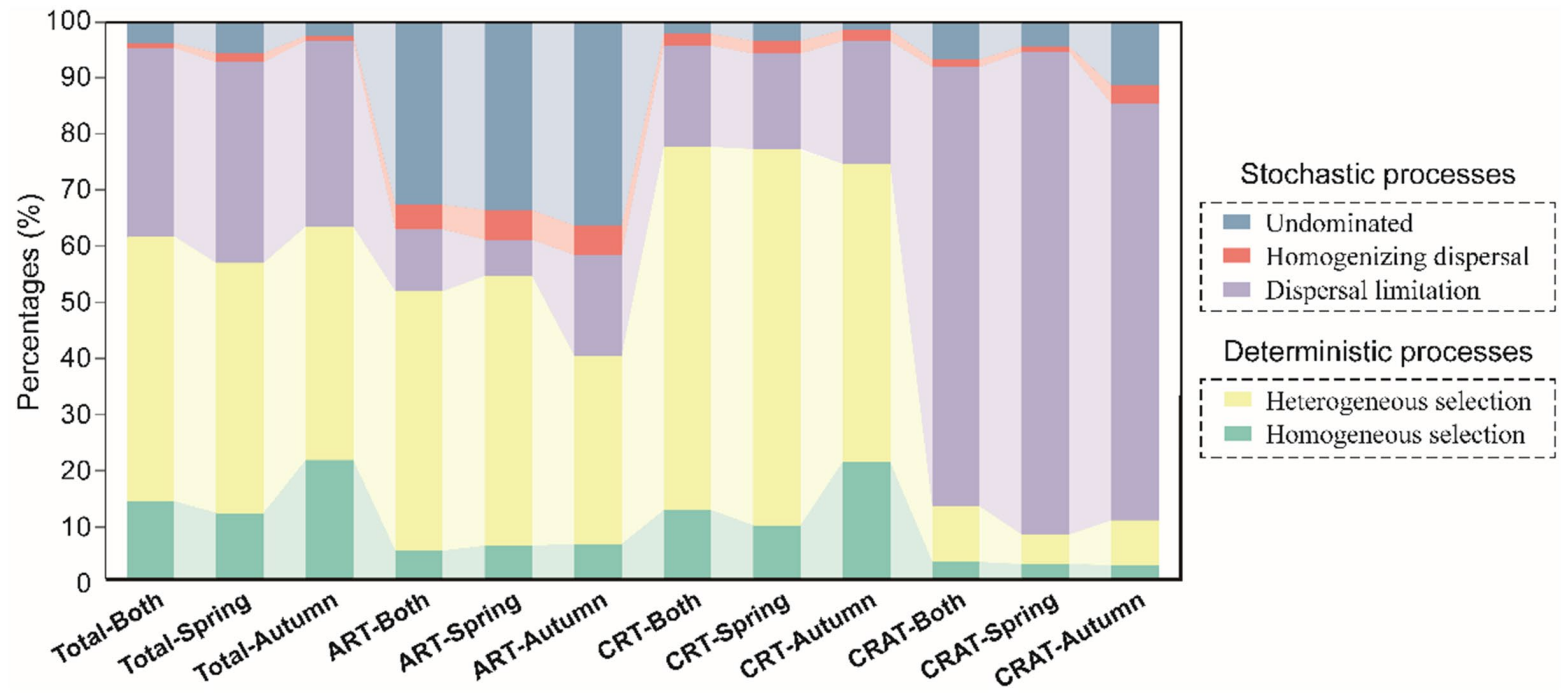
Community assembly mechanisms of sedimentary bacterial ART, CRT, and CRAT in spring and autumn. The barplot shows the relative contributions of stochastic processes (homogenizing dispersal, dispersal limitation, and undominated processes) and deterministic processes (heterogeneous selection and homogenous selection) in shaping sedimentary bacterial communities in spring and autumn.

The ecological processes shaping the community assembly were inferred based a combination of βNTI and RCbray metrics. Our analysis revealed distinct assembly mechanisms for the ART, CRT and CRAT groups (Figure 4). Specifically, stochastic processes predominated in the assembly of CRAT (87.0–92.1%), and deterministic processes played a dominant role in the assembly of CRT (74.7–77.6%). For ART, both stochastic (45.5–59.9%) and deterministic (40.1–54.4%) processes contributed significantly. Within the deterministic processes, heterogeneous selection emerged as the primary driver for CRT (53.7–67.7%). In contrast, dispersal limitation was the primary stochastic process influencing the CRAT assembly (74.9–86.8%). For ART, the assembly processes were more balanced, with the undominated stochastic (32.7–36.5%) and heterogeneous selection processes (33.9–48.5%) being the most prevalent. For the overall sedimentary bacterial community, deterministic processes had a dominant influence, with heterogeneous selection accounting for 42.0–47.7%, followed by dispersal limitation (33.4–36.1%), homogeneous selection (11.8–21.3%), undominated stochasticity (0.02–0.06%), and homogenizing dispersal (0.01%).

To explore the relationships between environmental factors and community assembly processes, we analyzed nine environmental factors: water temperature (WT), pH, ammonium nitrogen (NH_3_-N), nitrate nitrogen (NO_3_-N), total nitrogen (TN), total organic carbon (TOC), total phosphorus (TP), channel slope, and river flow. The longitudinal distributions of these parameters across the sediment at 24 stations along the Yangtze River are illustrated in Supplementary Figure S5A. Using Mantel and partial Mantel tests, we assessed the Spearman correlations between these environmental factors and the βNTI (Table 1; Supplementary Table S5). The Mantel results indicated that river flow was the sole factor significantly and positively associated with the βNTI for CRAT, which was observed only in spring. Additionally, WT, pH, TOC and river flow were significantly and positively correlated with the βNTI for CRT in both seasons, whereas they were significantly and positively associated with the βNTI of ART only in autumn (*p* < 0.05) (except TOC in both seasons). Notably, the partial Mantel tests showed that TOC was significantly and positively correlated with the βNTI of CRT in both seasons, and the βNTI of ART in spring.

**Table 1:** Mantel tests between the environmental factors and βNTI matrices of ART, CRT, and CRAT in the Yangtze River sediments for both spring and autumn seasons.

Environmental factors	ART (Mantel’s *r*)		CRT (Mantel’s *r*)		CRAT (Mantel’s *r*)	
	Spring	Autumn	Spring	Autumn	Spring	Autumn
NH_3_-N	**0.193** ^*^	0.130	0.107	0.112	−0.121	−0.022
NO_3_-N	−0.018	−0.101	0.020	−0.042	−0.017	0.107
TN	0.126	0.100	0.036	**0.115** ^*^	−0.074	−0.053
TOC	**0.135** ^*^	**0.161** ^*^	**0.171** ^**^	**0.178** ^**^	−0.028	−0.115
TP	0.020	0.122	0.122	0.002	0.035	0.010
WT	0.059	**0.145** ^*^	**0.095** ^*^	**0.121** ^*^	0.046	0.071
pH	0.086	**0.188** ^***^	**0.194** ^***^	**0.209** ^**^	0.057	0.011
River flow	0.037	**0.179** ^**^	**0.078** ^*^	**0.248** ^***^	**0.084** ^*^	0.036
Channel slope	0.034	**0.173** ^*^	**0.153** ^*^	0.042	0.055	−0.031

Asterisks and bold values denote the significance of statistics (^*^p < 0.05, ^**^p < 0.01, and ^***^p < 0.001). Bold values indicates *p* < 0.05.

### 3.5 Impacts of environmental factors on the sedimentary community

The Wilcoxon rank-sum tests revealed significant disparities in five environmental factors between spring and autumn (Supplementary Figure S5B). TOC and TP concentrations were lower in autumn than in spring, and WT, TN, and river flow displayed higher values during the autumn season (*p* < 0.05). Kruskal–Wallis tests further revealed that landform types had effects on specific environmental factors such as pH, NH_3_-N, NO_3_-N, river flow, and channel slope (*p* < 0.05) (Supplementary Figure S5C).

**Figure 5:**
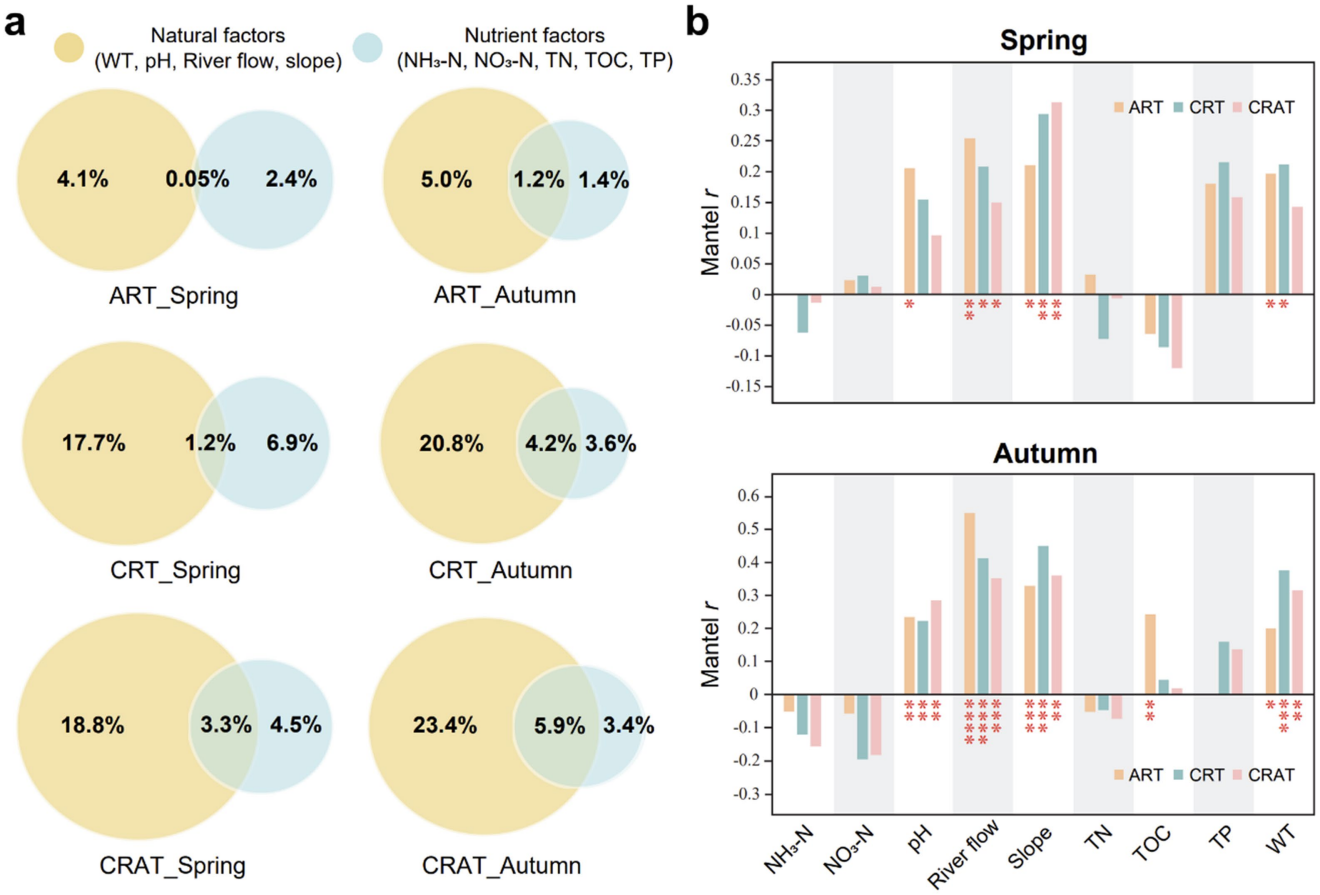
Environmental drivers of sedimentary bacterial ART, CRT, and CRAT community variations in spring and autumn. **(A)** VPA decoupling the effects of natural and nutrient factors on the community variations of ART, CRT, and CRAT. Natural factors include WT, pH, river flow, and channel slope. Nutrient factors include NH_3_-N, NO_3_-N, TN, TOC, and TP. **(B)** Mantel tests showing the Spearman correlations between environmental factors and community variations of ART, CRT, and CRAT in spring and autumn (^****^p < 0.0001, ^***^0.0001 < *p* < 0.001, and ^*^0.01 < *p* < 0.05, permutation = 9,999).

The environmental factors were categorized into two types: natural factors (WT, pH, river flow, channel slope) and nutrient factors (NH_3_-N, NO_3_-N, TN, TOC, TP), to assess their influence on community variations in different seasons (Figure 5A). Our results showed that natural factors had a greater impact than nutrient factors on changes in the structure of abundant and rare communities. Among the ART, CRT and CRAT, the CRAT community structure was most significantly influenced by both types of environmental factors, followed by CRT and ART, indicating that richer taxa were more susceptible to the environmental drivers. Moreover, during autumn, the pure and overall influence of natural factors on the variations of ART, CRT, and CRAT were greater than those in spring, and the pure impacts of nutrient factors were diminished compared with those in spring.

The Mantel Spearman correlations between the environmental factors and the bacterial community further confirmed that the community structures of ART, CRT, and CRAT in autumn were more strongly affected by environmental factors (Figure 5B). In both seasons, river flow and channel slope had significant effects on all three sub-communities (ART, CRT and CRAT) (Mantel *r* = 0.14–0.55, *p* < 0.05). Additionally, WT significantly affected ART and CRT in both seasons (Mantel *r* = 0.20–0.38, *p* < 0.05), ART was also influenced by pH in spring and autumn (Mantel *r* = 0.21, *p* < 0.05; Mantel *r* = 0.23, *p* < 0.01) and by TOC in autumn (Mantel *r* = 0.24, *p* < 0.01). CRT was influenced by pH in autumn (Mantel *r* = 0.22, *p* < 0.01), and for CRAT, responses to pH and WT were evident in autumn (Mantel *r* = 0.29, *p* < 0.01; Mantel *r* = 0.31, *p* < 0.01).

## 4 Discussion

The Yangtze River, the longest river in Asia, traverses China’s most economically vibrant provinces (Wang et al., 2019). Therefore, effective management of water pollution in the Yangtze River is paramount for ensuring the sustainable development of the Yangtze River economic belt. Given the heightened sensitivity of microbial communities to alterations in pH, nitrogen, phosphorus, and other nutrients or pollutants in freshwater sediments, these communities have emerged as crucial indicators for assessing water quality (Luo et al., 2024; Wu et al., 2019). Recent investigations into various riverine microorganisms have been conducted by Chen et al. (2019), Liu et al. (2018, 2023), Lv et al. (2021), Wang et al. (2020), and Yi et al. (2022). Liu et al. (2018) pioneered the analysis of spatiotemporal patterns of planktonic and sedimentary bacterial communities in the Yangtze River, and highlighted the pivotal role of benthic bacteria in contributing to riverine bacterial diversity. Our study further explored the differential biogeographic patterns and assembly mechanisms of benthic bacterial ART, CRT, and CRAT and linked their spatiotemporal dynamics to environmental gradients, which can provide important guidance for water resource management in large rivers.

### 4.1 Significant spatiotemporal distribution of microorganisms in river sediment

The alpha diversity analysis (Figure 1) of the benthic bacterial communities in the Yangtze River revealed intriguing seasonal dynamics, with higher diversity and richness indices observed in autumn than in spring. This finding was consistent with the observed patterns in microeukaryotic plankton and planktonic bacteria (Chen et al., 2019; Yi et al., 2022), suggesting that seasonal shifts played a pivotal role in shaping microbial communities in the river. Furthermore, NMDS and ANOSIM confirmed the distinct compositions of benthic bacterial communities between spring and autumn (Supplementary Figure S3). These differences can be attributed to seasonal variations in physical and chemical factors such as temperature, TN, TP, and river flow, which exhibited significant differences between the seasons (*p* < 0.05) (Supplementary Figure S5). In autumn, the elevated temperature provided favorable conditions for microbial proliferation, whereas increasing water levels and flow velocities may have led to bacterial dilution within sedimentary environments, impacting bacterial diversity either positively or negatively (Zhang et al., 2019).

To examine the influence of spatial distribution, the study area was divided into five distinct landform types. Our findings emphasized the crucial role of terrain and landforms in shaping community structure (Figure 2), which aligns with previous studies that suggested similar ecological niche partitioning in the Yangtze River (Liu et al., 2018; Yi et al., 2022). Several factors may contribute to these observations: first, there were significant differences in two-thirds of the physical and chemical parameters among the different landform types (*p* < 0.05), indicating environmental stress across these regions (Supplementary Figure S5); second, there was a significant geographical decay in the microbial community similarity in both seasons, indicating spatial heterogeneity across regions (Figure 2); and third, varying levels of human pollution were found across geographical regions, particularly affecting water resources in the middle and lower reaches of river basin (Wu et al., 2024). Overall, our study highlighted the complex interplay between temporal and spatial factors in influencing benthic bacterial communities in the Yangtze River. While attributing the spatiotemporal variations of benthic bacteria in large rivers to specific individual parameters remains challenging (Wang et al., 2020), our findings provide valuable insights into the dynamics of microbial communities and their potential responses to environmental changes.

### 4.2 Biogeographical differences between abundant and rare communities

Numerous studies have underscored the pivotal role of rare taxa in driving ecosystem dynamics (Lynch and Neufeld, 2015; Wan et al., 2021b; Zhu et al., 2022). To gain deeper insight into the dynamics and functions of microbial communities, we conducted systematic differentiation analyses between rare and abundant taxa. NMDS and ANOSIM revealed significant seasonal variations in the community structures of ART, CRT, and CRAT (*p* = 0.001). Notably, ART and CRT exhibited higher alpha diversity in autumn than in spring, suggesting that seasonal changes had a pronounced impact on the diversity of these groups. Additionally, ART and CRT presented greater beta diversity than CRAT, and both CRT and CRAT presented similar distance decay patterns, with ART being the least affected by geographical distance, implying potentially greater stability of the rarest communities in the environment. Furthermore, landforms significantly influenced all three groups, with ANOSIM indicating that the community differences in spring (*r* = 0.41–0.55) were greater than those observed in autumn (*r* = 0.26–0.54). This disparity may be attributed to the flushing of river sediments during autumn, which could facilitate the dispersal of certain benthic bacteria. In summary, both the abundant and rare communities exhibited notable spatial and temporal differences (Figures 1, 2; Supplementary Figure S3). Our findings are aligned with recent studies that revealed analogous biogeographic distribution patterns for both abundant and rare species in various ecosystems, such as the Taihu Lake basin, coastal Antarctic lakes, Ting River, and Yangtze River (Chen et al., 2019; Logares et al., 2013; Yi et al., 2022; Zhang T. et al., 2022).

According to VPA, the collective influence of all the environmental factors on the community structure was found to be under 35%, indicating a relatively minor role of these factors in shaping the community (Figure 5). Among these factors, natural factors (river flow and channel slope) had more pronounced effects than nutrient factors on the community structure. However, despite its frequent application for discerning the effects of ecological processes, it is essential to acknowledge that this approach might underestimate the true influence of environmental factors on the community structure (Gilbert and Bennett, 2010; Smith and Lundholm, 2010), suggesting that it should not serve as the sole method for understanding community dynamics.

### 4.3 Rare taxa dominate the nonrandom co-occurrence network of benthic bacteria

To elucidate the intricate roles of abundant and rare taxa in shaping the structure and dynamics of microbial communities, we analyzed co-occurrence networks within benthic bacterial communities in the Yangtze River (Wang et al., 2017). Understanding these interactions is critical for elucidating the underlying processes that govern microbial communities (Chen et al., 2023; Li et al., 2023; Jiao et al., 2022). In our study, the co-occurrence network of the benthic bacterial communities presented nonrandom characteristics and a modular structure, which aligns with previous findings (Wang et al., 2020; Xue et al., 2018). Key species are typically considered as crucial contributors, fulfilling indispensable roles within communities (Hu et al., 2017; Wang et al., 2020). Our analysis identified 136 key species (Supplementary Table S3), with the overwhelming majority (132, 97.05%) classified as CRT and only a small fraction (4, 2.95%) as CRAT, indicating strong interactions within the network. These findings suggest the significant influence of CRT on other communities and their crucial role in the overall community assembly. The prevalence of CRT as key species highlights the importance of rare taxa in microbial ecosystems, as they have been shown to exhibit highly active metabolisms and play a pivotal role in regulating aquatic ecosystem functions (Lynch and Neufeld, 2015; Wu et al., 2023). Furthermore, rare taxa serve as reservoirs of genetic diversity, which is essential for the resilience and stability of ecosystems (Galand et al., 2009; Jia et al., 2018). Thus, the pronounced influence of rare communities on the composition and structure of the entire sediment bacterial community, compared with the influence of abundant taxa, is consistent with the results of previous investigations of riverine bacterial communities (Wang et al., 2020; Wu et al., 2023).

Within the co-occurrence network, Acidobacteriota ASVs emerged as central players, exhibiting the highest abundance and richness among the keystone species. Even with relatively low abundance, Planctomycetota occupied a key position within the network, as evidenced by their high node degree, betweenness centrality and closeness centrality values in the topological analysis (Supplementary Figure S4). The high O/R ratios >1 between Acidobacteriota and Planctomycetota and other dominant taxa underscore the crucial role of interspecies associations in community assembly and function (Liu et al., 2023). While our approach offered valuable insights into potential species associations, it is important to note that it merely reflects statistical correlations and may not accurately reflect true ecological interactions. Moreover, correlation-based methods are also susceptible to both false positives and negatives, particularly when indirect relationships exist between species.

### 4.4 Differences in community assembly processes between abundant and rare bacterial taxa

The exploration of the fundamentally different community assembly mechanisms between abundant and rare bacteria is a key area of ecological research, with significant implications for understanding microbial community dynamics (Jia et al., 2018; Wang et al., 2022). Null model analysis indicated that the abundant taxa (CRAT) in the Yangtze River benthic bacteria were predominantly influenced by stochastic processes (92.1% in spring and 89.5% in autumn), whereas the rare taxa (ART/CRT) were more influenced by deterministic processes (65.9% in spring and 57.4% in autumn) (Figure 4). Further examination revealed that the assembly of CRAT was predominantly governed by dispersal limitation, whereas that of CRT was primarily controlled by heterogeneous selection. For ART, both heterogeneous selection and undominated stochastic processes jointly governed their assembly (Figure 4). These findings are generally consistent with observations made in various ecosystems, including coastal wetland soils (Gao et al., 2020; Yang et al., 2022), saline agricultural soils (Wan et al., 2021c), soils in arid inland river basin (Wang et al., 2022) and lakes (Wan et al., 2021b). However, they contrast sharply with results from the subsurface layer of marginal seas (Wu et al., 2017) and Tibetan Plateau soil (Ji et al., 2020), where stochastic processes were found to significantly impact both abundant and rare taxa, suggesting that the study scale and habitat might influence these outcomes (Shi et al., 2018; Yang et al., 2022; Yi et al., 2022).

The observed differences in assembly processes between the abundant and rare communities could be attributed to disparities in ecological niche occupation and tolerance capabilities. Abundant taxa, which occupy broad ecological niches, typically demonstrate enhanced resistance to environmental changes and have limited diffusion opportunities due to dispersal limitation, aligning with the steep distance-decay slope (Figure 2B) (Yang et al., 2022). In contrast, rare taxa, with narrow ecological niches, encounter increased environmental selection pressures (Wang et al., 2022; Zhang et al., 2021; Zhang T. et al., 2022). This inclination led to a high prevalence of deterministic processes in the assembly of ART and CRT. The critical role of the undominated stochastic and homogenizing dispersal process in the assembly of ART may be the reason for its least distance-decay slope.

Our research revealed that the community assembly of benthic bacteria in the Yangtze River was primarily influenced by deterministic processes (62%) (Figure 4). This finding contrasted with previous studies in the same basin, which indicated that planktonic bacteria were primarily driven by stochastic processes (Liu et al., 2018; Yi et al., 2022). This divergence can be attributed to the dynamic river water environment and spatiotemporal variability of resources, which impacted the planktonic community more significantly than the benthic community. Benthic bacteria, which reside in relatively stable environments, are substantially influenced by persistent environmental factors, such as organic matter content and redox gradients, leading to a high contribution of deterministic processes (Chen et al., 2017).

Furthermore, our analysis revealed a strong association between the βNTI of rare taxa and TOC in both spring and autumn, underscoring the critical role of TOC in modulating the balance between deterministic and stochastic processes in community assembly for ART and CRT (Table 1). A recent study demonstrated that soil labile organic carbon fractions mediate microbial community assembly processes during long-term vegetation (Shi et al., 2023). Elevated TOC levels could intensify inter-community competition, thereby augmenting the influence of deterministic processes on assembly (Qiu et al., 2023). Concurrently, TOC fluctuations could impact the dispersal capabilities and environmental adaptability of benthic bacteria, modifying the influence of stochastic processes in the community (Zhu et al., 2024). Consequently, TOC levels were instrumental in determining the equilibrium between deterministic and stochastic assembly processes in the Yangtze River benthic bacterial communities.

### 4.5 Implications for environmental management

Microbial diversity in river ecosystems serves as a sensitive indicator of environmental changes and can provide valuable insights into the effectiveness of ecological restoration efforts. Our study underscores the profound influence of natural factors on the sedimentary bacterial community structure in the Yangtze River, surpassing the effects of nutrients such as nitrogen and phosphorus (Figure 5). This highlights the importance of maintaining the river’s natural state to preserve microbial diversity and community function. Natural factors directly impact the living conditions and community structure of aquatic microbes. For instance, flow rate variations can influence microorganism distributions and dissolved oxygen levels, and changes in channel slope influence flow patterns and biological habitats. The benthic bacterial co-occurrence network in the Yangtze River was dominated by keystone species such as Acidobacteria, Proteobacteria, Planctomycetes, Bacteroidetes and Chloroflexi (Figure 3). These taxa may be intimately tied to environmental conditions. For instance, the relative abundances of Acidobacteria and Actinobacteria, which are slow-growing oligotrophic bacteria, tend to decrease as pollution levels increase (Zhang H. et al., 2022). In contrast, Proteobacteria, which are considered eutrophic bacteria, thrive in nutrient-rich environments. Chloroflexi contain aerobic chemoheterotrophs or anaerobic photoheterotrophs capable of decomposing organic matter and releasing inorganic nitrogen and phosphorus, making them suitable for nutrient-rich environments (Ma et al., 2023). The absence or low abundance of certain class *Betaproteobacteria* as well as genera like *Geobacter* and *Trichococcus* are typically found in metal-contaminated environments, implying good overall water quality of the Yangtze River (Gupta et al., 2023; Zhang et al., 2020). O/R values >1 between many key phyla indicated widespread co-occurrence patterns, suggesting that benthic bacteria maintain community stability through multiple interactions. Strong interactions among oligotrophic bacteria may also facilitate efficient nutrient utilization, contributing to the overall health of aquatic ecosystems (Gupta et al., 2023). Given the significance of the Yangtze River as the largest river in Asia, it is imperative to prioritize the conservation and protection of its natural state and ecological functions. This includes minimizing human disturbances and damage to the river, as well as preserving the ecological integrity of river channels to maintain the health and stability of the aquatic ecosystem.

## 5 Conclusion

This comprehensive study explored the intricate spatial distribution, coexistence patterns, and assembly processes of abundant and rare taxa within the sediment of the Yangtze River, the third-largest river in the world, which provides crucial information for understanding the community structure and composition of riverine microorganisms. The results demonstrated significant seasonal variations in community structure across various taxa, and highlighted the profound influence of spatial and environmental factors on the distribution of sedimentary microorganisms. ART/CRT contributed greatly to higher alpha diversity in autumn than in spring. The CRT community emerged as a key player represented by Acidobacteria, exerting a pivotal influence on the sedimentary ecosystem dynamics through potential inter-microbial interactions. CRT and CRAT exhibited more pronounced distance decay patterns than ART. Further analysis revealed distinct assembly mechanisms governing the various benthic bacterial communities: dispersal limitation was the dominant process for the CRAT community, heterogeneous selection primarily influenced the CRT community, and the ART community was shaped by a combination of heterogeneous selection and undominated stochastic processes. Given the prevalence and abundance of CRT in the overall community, the assembly mechanism of CRT likely reflects the entire taxonomic group. However, it is important to acknowledge that benthic bacterial communities were investigated at specific time points (spring and autumn) in this study, which may not have fully captured the long-term dynamics of the communities. To gain a more comprehensive understanding of the intricate long-term trends in microbial community dynamics, future researches should significantly increase the sampling frequency, encompassing a broader range of seasons and spanning multiple years.

## Data Availability

The original contributions presented in the study are included in the article/Supplementary material, further inquiries can be directed to the corresponding author.

## Footnotes

^1^ https://gephi.org/
