# Supplementary material for: Spatiotemporal distributions, co-occurrence networks, and assembly mechanisms of the bacterial community in sediments of the Yangtze River: comprehensive insights into abundant and rare taxa
Source: Front Microbiol. 2024 Dec 11;15:1444206. doi: 10.3389/fmicb.2024.1444206 (PMC11668926; doi:10.3389/fmicb.2024.1444206)
Supplement: Supplementary file 1 [file Data_Sheet_1.docx]

**Supplementary Information**

**Spatiotemporal distributions, co-occurrence networks, and assembly mechanisms of the bacterial community in sediments of the Yangtze River: comprehensive insights into abundant and rare taxa**

**Authors**: Guohua Zhang^1^, Shufeng Liu^1,2^*, Wenran Du^3^, Yinghao Li^1^, Zongzhi Wu^1^, Tang Liu^4^, Yichu Wang^5^

**Author affiliations**:

^1^Key Laboratory of Water and Sediment Sciences, Ministry of Education, College of Environmental Sciences and Engineering, Peking University, Beijing, P.R. China

^2^College of Resources and Environmental Sciences, China Agricultural University, Beijing, P.R. China

^3^School of Environment and Energy, Peking University Shenzhen Graduate School, Shenzhen, P.R. China

^4^College of Chemistry and Environmental Engineering, Shenzhen University, Shenzhen, P.R. China

^5^College of Water Sciences, Beijing Normal University, Beijing, P.R. China

***Corresponding author:** Shufeng Liu

Postal address: China Agricultural University, No. 2 Yuanmingyuan West Road, Beijing 100193, P.R. China

E-mail address: liushufeng@cau.edu.cn

**
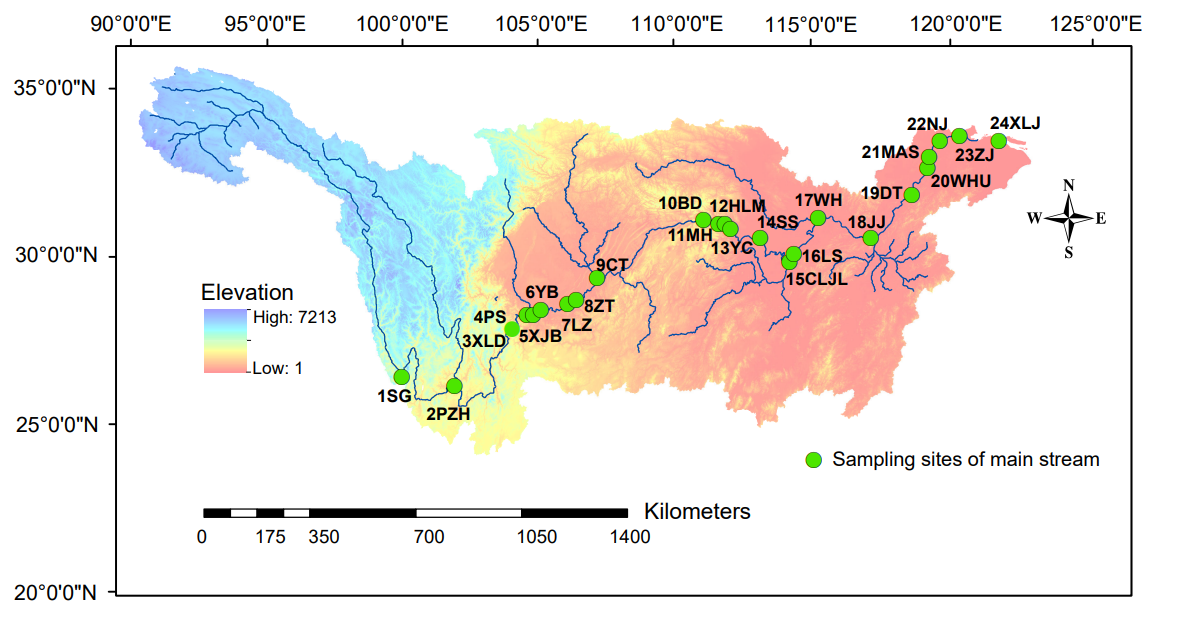
**

**Figure S1.** Distribution of 24 sampling sites along the Yangtze River mainstream. Detailed information about the sampling sites is listed in Table S1.


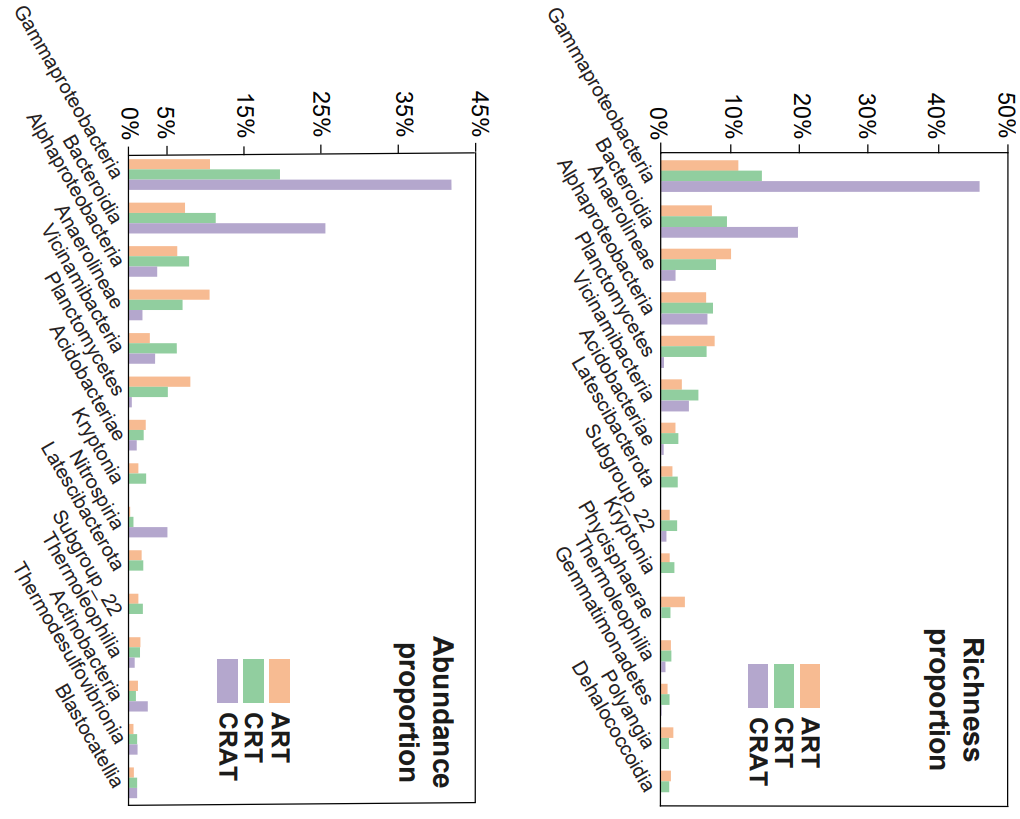


**Figure S2.** Richness and abundance proportions in bacterial ART, CRT, and CRAT at class level in the Yangtze River sediments. Only the top 15 richest or most abundant classes are shown.


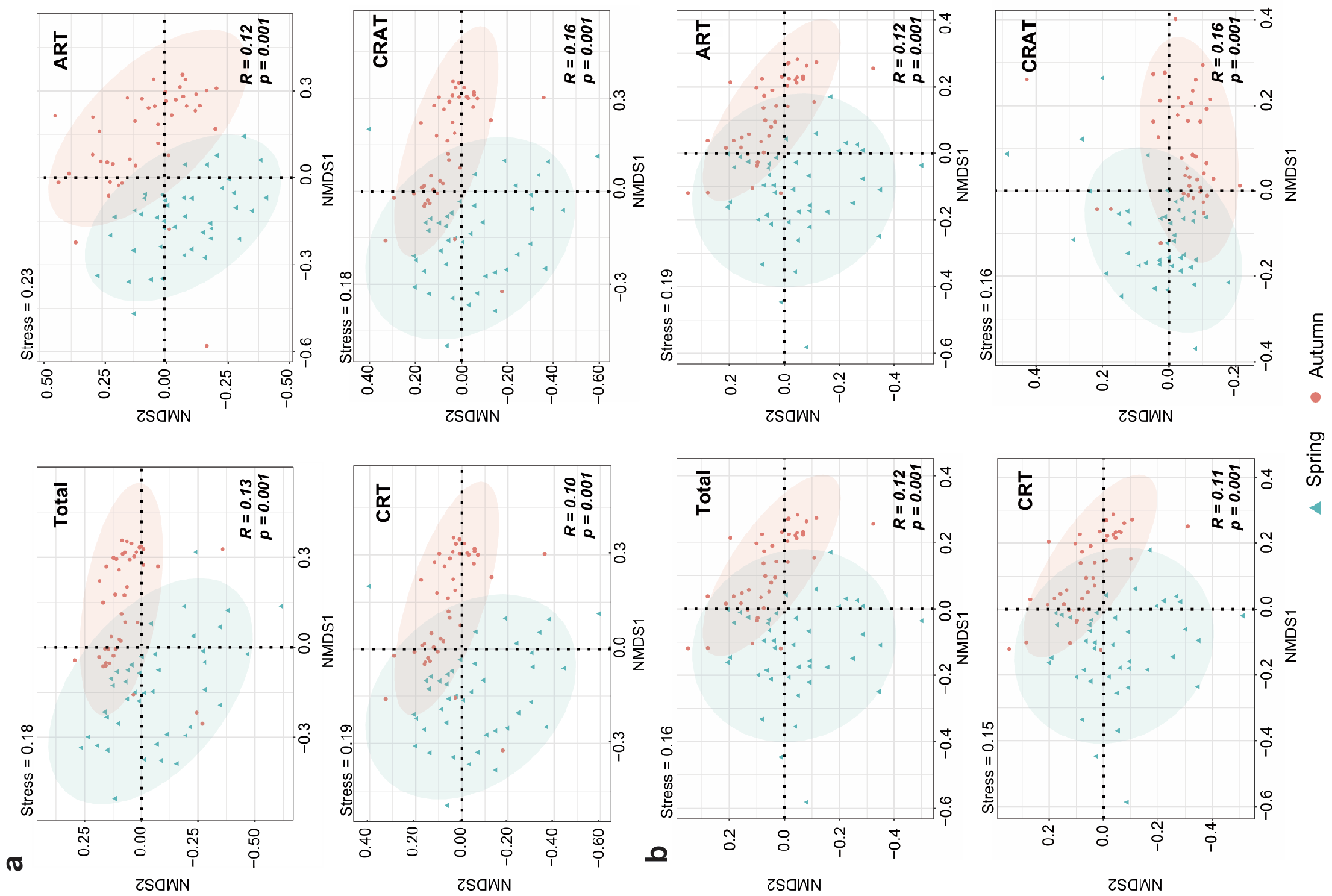


**Figure S3.** Differences in community structure of total bacteria, ART, CRT, and CRAT between spring and autumn in the Yangtze River sediments, as revealed by NMDS and ANOSIM based on Bray-Curtis (**a**) and unweighted Unifrac (**b**) dissimilarities.


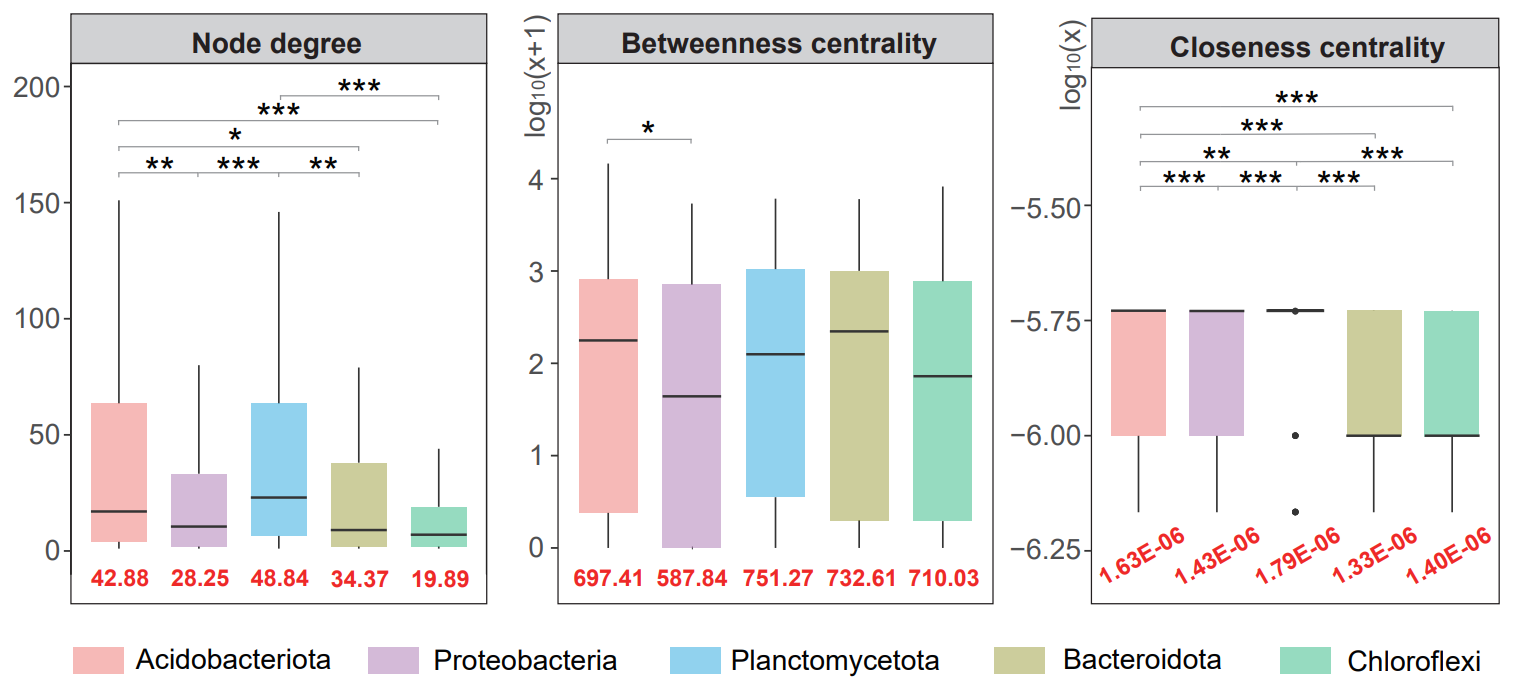


**Figure S4.** Comparisons of the typical node-level topological features among the five phyla with the most ASVs involved in the sedimentary bacterial co-occurrence network. Asterisks denote the significance of statistical tests (Wilcoxon rank-sum tests: ***0.0001 < *p* < 0.001, **0.001 < *p* < 0.01, and *0.01 < *p* < 0.05). On the bottom of each boxplot, the mean values are provided.


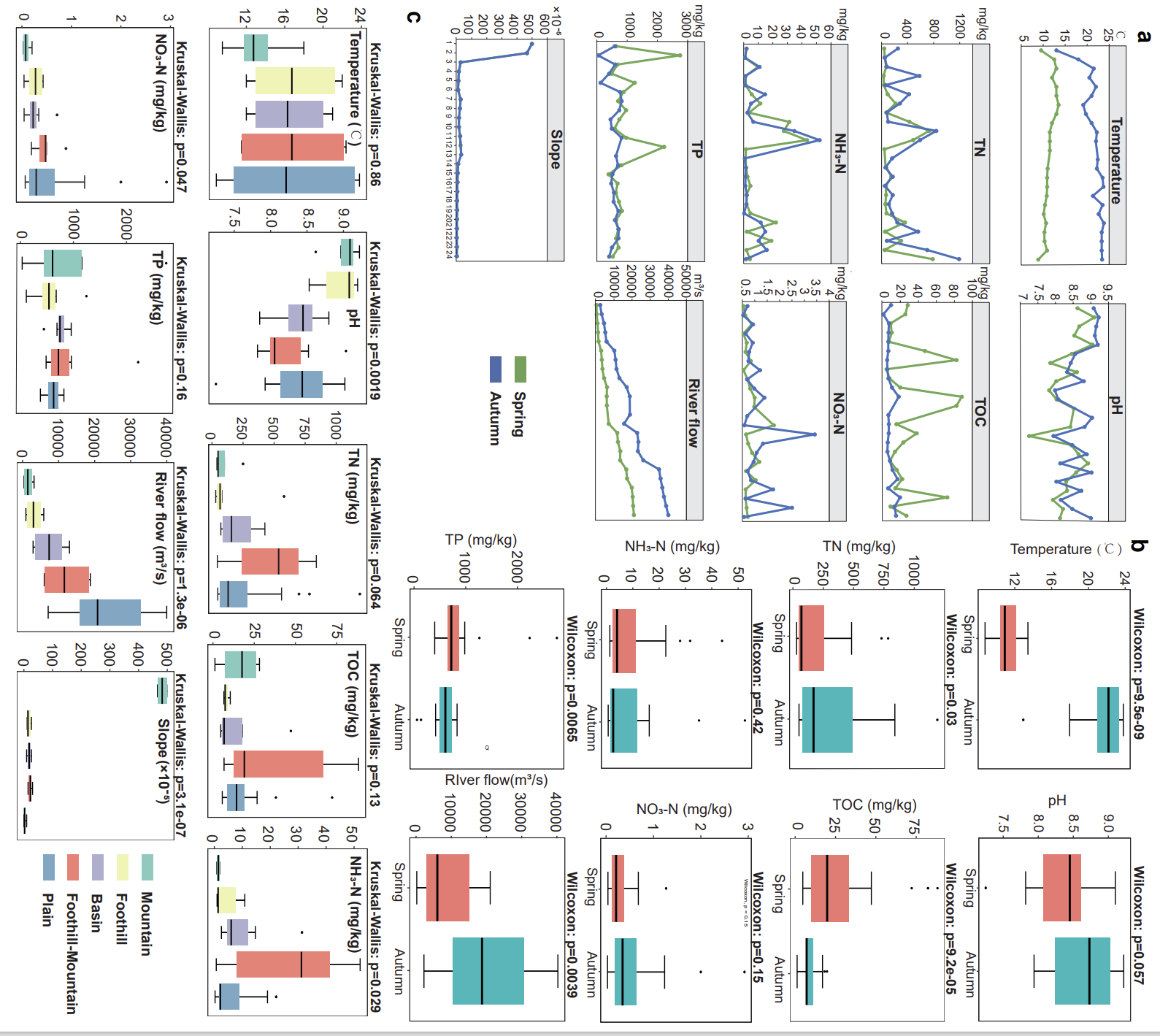


**Figure S5.** Spatial and temporal variations of the environmental factors in sediments of the Yangtze River. (**a**) Line charts showing how these environmental factors vary from site to site in spring and autumn. (**b**) Wilcoxon rank-sum tests for the difference between spring and autumn. (**c**) Kruskal-Wallis tests for the difference among the five landform types.

**Table S1**. Detailed information of the 24 sampling sites along the Yangtze River.

| **Site No.** | **Full name** | **Abbreviation** | **Longitude (°E)^a^** | **Latitude (°N)^a^** | **Landforms** | **Group** |
| --- | --- | --- | --- | --- | --- | --- |
|  |  |  |  |  |  |  |
| 1 | ShiGu | SG | 99.98 | 26.88 | Mountain | Upstream |
| 2 | PanZhiHua | PZH | 101.70 | 26.57 | Mountain | Upstream |
| 3 | XiLuoDu | XLD | 103.66 | 28.25 | Foothill | Upstream |
| 4 | PingShan | PS | 104.17 | 28.65 | Foothill | Upstream |
| 5 | XiangJiaBa | XJB | 104.38 | 28.65 | Foothill | Upstream |
| 6 | YiBin | YB | 104.65 | 28.77 | Basin | Upstream |
| 7 | LuZhou | LZ | 105.55 | 28.90 | Basin | Upstream |
| 8 | ZhuTuo | ZT | 105.85 | 29.02 | Basin | Upstream |
| 9 | CunTan | CT | 106.60 | 29.62 | Basin | Upstream |
| 10 | BaDong | BD | 110.40 | 31.04 | Mountain-Foothill | Upstream |
| 11 | MiaoHe | MH | 110.90 | 30.88 | Mountain-Foothill | Upstream |
| 12 | HuangLingMiao | HLM | 111.12 | 30.85 | Mountain-Foothill | Upstream |
| 13 | YiChang | YC | 111.28 | 30.69 | Mountain-Foothill | Upstream |
| 14 | ShaShi | SS | 112.26 | 30.29 | Plain | Midstream |
| 15 | ChengLingJiLian | CLJL | 113.15 | 29.45 | Plain | Midstream |
| 16 | LuoShan | LS | 113.32 | 29.67 | Plain | Midstream |
| 17 | WuHan | WH | 114.32 | 30.62 | Plain | Midstream |
| 18 | JiuJiang | JJ | 116.00 | 29.74 | Plain | Midstream |
| 19 | DaTong | DT | 117.64 | 30.78 | Plain | Downstream |
| 20 | WuHu | WHU | 118.34 | 31.46 | Plain | Downstream |
| 21 | MaAnShan | MAS | 118.47 | 31.77 | Plain | Downstream |
| 22 | NanJing | NJ | 118.94 | 32.17 | Plain | Downstream |
| 23 | ZhenJiang | ZJ | 119.66 | 32.18 | Plain | Downstream |
| 24 | XuLiuJing | XLJ | 120.96 | 31.77 | Plain | Downstream |

^a^ The longitude and latitude of each site were determined by GPS.

**Table S2**. Good’s coverage of the overall sedimentary bacterial community in each site of the Yangtze River (including the nonrarefied and rarefied). For the replicated samples, the average value of coverage is given.

| **Site ID** | **Spring_nonrarefied** | **Autumn_nonrarefied** | **Spring_rarefied** | **Autumn_rarefied** |
| --- | --- | --- | --- | --- |
| 1SG | 0.99274235 | 0.991024747 | 0.94412091 | 0.943600943 |
| 2PZH | 0.996668854 | 0.992572675 | 0.968074043 | 0.92900721 |
| 3XLD | 0.994839864 | 0.990801158 | 0.971575153 | 0.926095397 |
| 4PS | 0.99514383 | 0.996015523 | 0.965890183 | 0.953272324 |
| 5XJB | 0.993919383 | 0.992337629 | 0.958056018 | 0.943219634 |
| 6YB | 0.987064579 | 0.986922242 | 0.92113838 | 0.891950915 |
| 7LZ | 0.988068862 | 0.99600058 | 0.944005361 | 0.958056018 |
| 8ZT | 0.98600536 | 0.984314129 | 0.953618968 | 0.901656961 |
| 9CT | 0.995392233 | 0.981024007 | 0.960031891 | 0.867963117 |
| 10BD | 0.98155055 | 0.978056205 | 0.937881309 | 0.893372158 |
| 11MH | 0.985204582 | 0.981051809 | 0.911721205 | 0.860995563 |
| 12HLM | 0.995164519 | NA | 0.96578619 | NA |
| 13YC | NA | 0.989677408 | NA | 0.933929562 |
| 14SS | 0.995508702 | 0.993121766 | 0.949944537 | 0.953653633 |
| 15CLJL | 0.983190395 | 0.985344617 | 0.938158625 | 0.944224903 |
| 16LS | 0.993978041 | 0.988840413 | 0.956496118 | 0.880615641 |
| 17WH | 0.993904395 | 0.993371149 | 0.937881309 | 0.948419301 |
| 18JJ | 0.986939918 | 0.983329476 | 0.9619731 | 0.910045757 |
| 19DT | 0.983865505 | 0.991471215 | 0.906475319 | 0.933860233 |
| 20WHU | 0.987905121 | 0.977044169 | 0.916909318 | 0.905227399 |
| 21MAS | 0.98468497 | 0.992054101 | 0.923460899 | 0.940099834 |
| 22NJ | 0.981157363 | 0.981434917 | 0.918711869 | 0.917498613 |
| 23ZJ | 0.984803675 | 0.983370921 | 0.903806156 | 0.948661952 |
| 24XLJ | 0.987787905 | 0.988909944 | 0.948627288 | 0.956345905 |

NA, dataset not available due to PCR failure or sample shortage.

**Table S3**. Taxonomic classification of the keystone ASVs in the co-occurrence network with their relative abundances in each landform type.

| **No.** | **Taxonomy** | **Category** | **Mean relative abundance (%)** | | | | |
| --- | --- | --- | --- | --- | --- | --- | --- |
|  |  |  | **Mountain** | **Foothill** | **Basin­** | **Foothill-Mountain** | **Plain** |
| 1 | d__Bacteria; p__Acidobacteriota; c__Acidobacteriae; o__Subgroup_2; f__Subgroup_2; g__Subgroup_2; s__uncultured_bacterium | CRT | 0 | 0 | 0 | 0.004556226 | 1.997762628 |
| 2 | d__Bacteria; p__Acidobacteriota; c__Vicinamibacteria; o__Vicinamibacterales; f__Vicinamibacteraceae; g__Vicinamibacteraceae | CRT | 0 | 0.003496135 | 0.007627469 | 0.070220266 | 1.678360666 |
| 3 | d__Bacteria; p__Acidobacteriota; c__Blastocatellia; o__Pyrinomonadales; f__Pyrinomonadaceae; g__RB41 | CRT | 0.001053451 | 0 | 0.000980439 | 0 | 1.746047192 |
| 4 | d__Bacteria; p__Acidobacteriota; c__Vicinamibacteria; o__Subgroup_17; f__Subgroup_17; g__Subgroup_17 | CRT | 0 | 0 | 0.008817972 | 0.092875641 | 1.6293013 |
| 5 | d__Bacteria; p__Acidobacteriota; c__Blastocatellia; o__Blastocatellales; f__Blastocatellaceae; g__Blastocatella; s__uncultured_bacterium | CRT | 0 | 0 | 0.006038323 | 0.038280098 | 1.664676865 |
| 6 | d__Bacteria; p__Acidobacteriota; c__Subgroup_22; o__Subgroup_22; f__Subgroup_22; g__Subgroup_22; s__uncultured_bacterium | CRT | 0 | 0 | 0 | 0 | 1.225388894 |
| 7 | d__Bacteria; p__Acidobacteriota; c__Thermoanaerobaculia; o__Thermoanaerobaculales; f__Thermoanaerobaculaceae; g__Thermoanaerobaculum; s__uncultured_bacterium | CRT | 0 | 0 | 0.008023121 | 0.010609499 | 0.949109322 |
| 8 | d__Bacteria; p__Acidobacteriota; c__Blastocatellia; o__Pyrinomonadales; f__Pyrinomonadaceae; g__RB41 | CRT | 0 | 0 | 0 | 0 | 0.846849731 |
| 9 | d__Bacteria; p__Acidobacteriota; c__Acidobacteriae; o__Subgroup_15; f__Subgroup_15; g__Subgroup_15; s__uncultured_bacterium | CRT | 0 | 0 | 0.001164706 | 0 | 0.788301384 |
| 10 | d__Bacteria; p__Acidobacteriota; c__Blastocatellia; o__Blastocatellales; f__Blastocatellaceae; g__JGI_0001001-H03; s__uncultured_bacterium | CRT | 0 | 0 | 0 | 0 | 0.773425167 |
| 11 | d__Bacteria; p__Acidobacteriota; c__Vicinamibacteria; o__Subgroup_17; f__Subgroup_17; g__Subgroup_17 | CRT | 0 | 0.0008518 | 0 | 0.0035365 | 0.736176512 |
| 12 | d__Bacteria; p__Acidobacteriota; c__Vicinamibacteria; o__Vicinamibacterales; f__Vicinamibacteraceae; g__Vicinamibacteraceae | CRT | 0 | 0 | 0.001960878 | 0.030787283 | 0.621438643 |
| 13 | d__Bacteria; p__Acidobacteriota; c__Vicinamibacteria; o__Vicinamibacterales; f__uncultured; g__uncultured | CRT | 0 | 0 | 0 | 0 | 0.646105448 |
| 14 | d__Bacteria; p__Acidobacteriota; c__Acidobacteriae | CRTv | 0 | 0 | 0.001164706 | 0.004420633 | 0.632291235 |

**Table S3**. Continued.

| **No.** | **Taxonomy** | **Category** | **Mean relative abundance (%)** | | | | |
| --- | --- | --- | --- | --- | --- | --- | --- |
|  |  |  | **Mountain** | **Foothill** | **Basin­** | **Foothill-Mountain** | **Plain** |
| 15 | d__Bacteria; p__Acidobacteriota; c__Subgroup_22; o__Subgroup_22; f__Subgroup_22; g__Subgroup_22; s__uncultured_Acidobacteriales | CRT | 0 | 0 | 0.011785886 | 0.01480769 | 0.583325845 |
| 16 | d__Bacteria; p__Acidobacteriota; c__Acidobacteriae; o__Subgroup_2; f__Subgroup_2; g__Subgroup_2 | CRT | 0 | 0 | 0.004658824 | 0 | 0.592837426 |
| 17 | d__Bacteria; p__Acidobacteriota; c__Subgroup_11; o__Subgroup_11; f__Subgroup_11; g__Subgroup_11; s__uncultured_bacterium | CRT | 0 | 0.001748797 | 0.001164706 | 0.005404549 | 0.588771872 |
| 18 | d__Bacteria; p__Acidobacteriota; c__Subgroup_22; o__Subgroup_22; f__Subgroup_22; g__Subgroup_22 | CRT | 0 | 0 | 0 | 0.004420633 | 0.573657151 |
| 19 | d__Bacteria; p__Acidobacteriota; c__Vicinamibacteria; o__Vicinamibacterales; f__uncultured; g__uncultured | CRT | 0 | 0 | 0 | 0.012377766 | 0.530036754 |
| 20 | d__Bacteria; p__Acidobacteriota; c__Vicinamibacteria; o__Vicinamibacterales; f__Vicinamibacteraceae; g__Vicinamibacteraceae; s__uncultured_bacterium | CRT | 0 | 0 | 0 | 0.008841266 | 0.49951565 |
| 21 | d__Bacteria; p__Acidobacteriota; c__Subgroup_22; o__Subgroup_22; f__Subgroup_22; g__Subgroup_22; s__uncultured_bacterium | CRT | 0 | 0 | 0 | 0.016879094 | 0.454666436 |
| 22 | d__Bacteria; p__Acidobacteriota; c__Vicinamibacteria; o__Subgroup_17; f__Subgroup_17; g__Subgroup_17 | CRT | 0 | 0 | 0 | 0.00948707 | 0.415176609 |
| 23 | d__Bacteria; p__Acidobacteriota; c__Subgroup_22; o__Subgroup_22; f__Subgroup_22; g__Subgroup_22; s__uncultured_proteobacterium | CRT | 0 | 0 | 0.009733953 | 0.005439316 | 0.401175868 |
| 24 | d__Bacteria; p__Acidobacteriota; c__Subgroup_22; o__Subgroup_22; f__Subgroup_22; g__Subgroup_22 | CRT | 0 | 0 | 0.007812396 | 0.015286367 | 0.392805109 |
| 25 | d__Bacteria; p__Acidobacteriota; c__Vicinamibacteria; o__Vicinamibacterales; f__Vicinamibacteraceae; g__Luteitalea | CRT | 0 | 0 | 0 | 0.008348022 | 0.370966106 |
| 26 | d__Bacteria; p__Acidobacteriota; c__Blastocatellia; o__Blastocatellales; f__Blastocatellaceae; g__Blastocatella | CRT | 0 | 0 | 0 | 0 | 0.361934766 |
| 27 | d__Bacteria; p__Acidobacteriota; c__Vicinamibacteria; o__Subgroup_17; f__Subgroup_17; g__Subgroup_17 | CRT | 0 | 0 | 0 | 0.004420633 | 0.347531455 |
| 28 | d__Bacteria; p__Acidobacteriota; c__Subgroup_22; o__Subgroup_22; f__Subgroup_22; g__Subgroup_22 | CRT | 0 | 0 | 0 | 0 | 0.305394581 |

**Table S3**. Continued.

| **ID** | **Taxonomy** | **Category** | **Mean relative abundance (%)** | | | | |
| --- | --- | --- | --- | --- | --- | --- | --- |
|  |  |  | **Mountain** | **Foothill** | **Basin­** | **Foothill-Mountain** | **Plain** |
| 29 | d__Bacteria; p__Acidobacteriota; c__Subgroup_22; o__Subgroup_22; f__Subgroup_22; g__Subgroup_22; s__uncultured_bacterium | CRT | 0 | 0 | 0 | 0.0035365 | 0.295248948 |
| 30 | d__Bacteria; p__Acidobacteriota; c__Subgroup_22; o__Subgroup_22; f__Subgroup_22; g__Subgroup_22; s__uncultured_bacterium | CRT | 0 | 0 | 0 | 0.005468903 | 0.279526355 |
| 31 | d__Bacteria; p__Acidobacteriota; c__Subgroup_22; o__Subgroup_22; f__Subgroup_22; g__Subgroup_22 | CRT | 0 | 0 | 0 | 0.005695273 | 0.262611556 |
| 32 | d__Bacteria; p__Acidobacteriota; c__Subgroup_5; o__Subgroup_5; f__Subgroup_5; g__Subgroup_5; s__uncultured_bacterium | CRT | 0 | 0.001748797 | 0 | 0.004420633 | 0.260890989 |
| 33 | d__Bacteria; p__Acidobacteriota; c__Subgroup_22; o__Subgroup_22; f__Subgroup_22; g__Subgroup_22; s__uncultured_bacterium | CRT | 0 | 0 | 0 | 0.006188865 | 0.257221366 |
| 34 | d__Bacteria; p__Acidobacteriota; c__Acidobacteriae; o__Subgroup_2; f__Subgroup_2; g__Subgroup_2; s__uncultured_soil | CRT | 0 | 0 | 0 | 0.007838263 | 0.240735873 |
| 35 | d__Bacteria; p__Acidobacteriota; c__Subgroup_18; o__Subgroup_18; f__Subgroup_18; g__Subgroup_18 | CRT | 0 | 0 | 0 | 0 | 0.214247224 |
| 36 | d__Bacteria; p__Acidobacteriota; c__Subgroup_22; o__Subgroup_22; f__Subgroup_22; g__Subgroup_22; s__uncultured_bacterium | CRT | 0 | 0 | 0 | 0 | 0.211991971 |
| 37 | d__Bacteria; p__Acidobacteriota; c__Subgroup_20; o__Subgroup_20; f__Subgroup_20; g__Subgroup_20; s__uncultured_bacterium | CRT | 0 | 0 | 0 | 0.002652748 | 0.205194329 |
| 38 | d__Bacteria; p__Acidobacteriota; c__Vicinamibacteria; o__Vicinamibacterales; f__Vicinamibacteraceae; g__Vicinamibacteraceae | CRT | 0 | 0 | 0 | 0.001769658 | 0.203970797 |
| 39 | d__Bacteria; p__Acidobacteriota; c__Vicinamibacteria; o__Subgroup_17; f__Subgroup_17; g__Subgroup_17 | CRT | 0 | 0 | 0 | 0 | 0.178703734 |
| 40 | d__Bacteria; p__Acidobacteriota; c__Vicinamibacteria; o__Subgroup_17; f__Subgroup_17; g__Subgroup_17 | CRT | 0 | 0 | 0 | 0 | 0.173593212 |
| 41 | d__Bacteria; p__Actinobacteriota; c__Thermoleophilia; o__Gaiellales; f__Gaiellaceae; g__Gaiella; s__uncultured_soil | CRT | 0 | 0 | 0.009797751 | 0.016798364 | 1.617155398 |
| 42 | d__Bacteria; p__Actinobacteriota; c__Acidimicrobiia; o__Actinomarinales; f__uncultured; g__uncultured | CRT | 0 | 0 | 0 | 0 | 1.324678485 |

**Table S3**. Continued.

| **No.** | **Taxonomy** | **Category** | **Mean relative abundance (%)** | | | | |
| --- | --- | --- | --- | --- | --- | --- | --- |
|  |  |  | **Mountain** | **Foothill** | **Basin­** | **Foothill-Mountain** | **Plain** |
| 43 | d__Bacteria; p__Actinobacteriota; c__RBG-16-55-12; o__RBG-16-55-12; f__RBG-16-55-12; g__RBG-16-55-12; s__uncultured_bacterium | CRT | 0 | 0 | 0 | 0.001769658 | 0.23946318 |
| 44 | d__Bacteria; p__Armatimonadota; c__Fimbriimonadia; o__Fimbriimonadales | CRT | 0 | 0 | 0 | 0 | 0.326411962 |
| 45 | d__Bacteria; p__Bacteroidota; c__Kryptonia; o__Kryptoniales; f__BSV26; g__BSV26; s__uncultured_bacterium | CRT | 0 | 0.005682966 | 0.00953331 | 0.009725365 | 2.726064962 |
| 46 | d__Bacteria; p__Bacteroidota; c__Kryptonia; o__Kryptoniales; f__BSV26; g__BSV26; s__uncultured_soil | CRT | 0 | 0 | 0.008817972 | 0.036092015 | 1.945705309 |
| 47 | d__Bacteria; p__Bacteroidota; c__Kryptonia; o__Kryptoniales; f__BSV26; g__BSV26; s__uncultured_Chlorobi | CRT | 0 | 0 | 0 | 0 | 1.452956392 |
| 48 | d__Bacteria; p__Bacteroidota; c__Kryptonia; o__Kryptoniales; f__BSV26; g__BSV26; s__uncultured_bacterium | CRT | 0 | 0 | 0.013169696 | 0.028801025 | 1.280912309 |
| 49 | d__Bacteria; p__Bacteroidota; c__Kryptonia; o__Kryptoniales; f__BSV26; g__BSV26 | CRT | 0 | 0 | 0 | 0 | 0.959924713 |
| 50 | d__Bacteria; p__Bacteroidota; c__Bacteroidia; o__Chitinophagales; f__Chitinophagaceae; g__Terrimonas | CRT | 0 | 0 | 0 | 0.025217937 | 0.887112645 |
| 51 | d__Bacteria; p__Bacteroidota; c__Kryptonia; o__Kryptoniales; f__BSV26; g__BSV26; s__uncultured_bacterium | CRT | 0 | 0 | 0.002492819 | 0.006834321 | 0.85717 |
| 52 | d__Bacteria; p__Bacteroidota; c__Kryptonia; o__Kryptoniales; f__BSV26; g__BSV26; s__uncultured_bacterium | CRT | 0 | 0 | 0 | 0.005695273 | 0.824278006 |
| 53 | d__Bacteria; p__Bacteroidota; c__Kryptonia; o__Kryptoniales; f__BSV26; g__BSV26; s__uncultured_bacterium | CRT | 0 | 0 | 0 | 0 | 0.685419214 |
| 54 | d__Bacteria; p__Bacteroidota; c__Kryptonia; o__Kryptoniales; f__BSV26; g__BSV26; s__uncultured_bacterium | CRT | 0 | 0 | 0.001164706 | 0 | 0.658721405 |
| 55 | d__Bacteria; p__Bacteroidota; c__Kryptonia; o__Kryptoniales; f__BSV26; g__BSV26 | CRT | 0 | 0 | 0 | 0.008466127 | 0.583033904 |
| 56 | d__Bacteria; p__Bacteroidota; c__Bacteroidia; o__Flavobacteriales; f__NS9_marine_group; g__NS9_marine_group; s__uncultured_bacterium | CRT | 0 | 0 | 0.0083095 | 0.018566631 | 0.438503724 |
| 57 | d__Bacteria; p__Bacteroidota; c__Kryptonia; o__Kryptoniales; f__BSV26; g__BSV26; s__uncultured_bacterium | CRT | 0 | 0 | 0.000584091 | 0.001769658 | 0.447366372 |

**Table S3**. Continued.

| **No.** | **Taxonomy** | **Category** | **Mean relative abundance (%)** | | | | |
| --- | --- | --- | --- | --- | --- | --- | --- |
|  |  |  | **Mountain** | **Foothill** | **Basin­** | **Foothill-Mountain** | **Plain** |
| 58 | d__Bacteria; p__Bacteroidota; c__Kryptonia; o__Kryptoniales; f__BSV26; g__BSV26; s__uncultured_bacterium | CRT | 0 | 0 | 0.002329412 | 0.006963899 | 0.393462385 |
| 59 | d__Bacteria; p__Bacteroidota; c__SJA-28; o__SJA-28; f__SJA-28; g__SJA-28; s__uncultured_Ignavibacterium | CRT | 0 | 0 | 0 | 0.002652748 | 0.392373229 |
| 60 | d__Bacteria; p__Bacteroidota; c__Ignavibacteria; o__Ignavibacteriales; f__Ignavibacteriaceae; g__Ignavibacterium; s__uncultured_bacterium | CRT | 0 | 0 | 0 | 0 | 0.174477554 |
| 61 | d__Bacteria; p__Chloroflexi; c__Anaerolineae; o__Anaerolineales; f__Anaerolineaceae; g__uncultured | CRT | 0 | 0 | 0.005824956 | 0.030347685 | 3.70536044 |
| 62 | d__Bacteria; p__Chloroflexi; c__Anaerolineae; o__Anaerolineales; f__Anaerolineaceae; g__uncultured | CRAT | 0 | 0 | 0.01130634 | 0.010609499 | 0.607825872 |
| 63 | d__Bacteria; p__Chloroflexi; c__Anaerolineae; o__Anaerolineales; f__Anaerolineaceae; g__uncultured; s__uncultured_Anaerolineaceae | CRT | 0 | 0 | 0 | 0 | 0.606277438 |
| 64 | d__Bacteria; p__Chloroflexi; c__Anaerolineae; o__Anaerolineales; f__Anaerolineaceae; g__uncultured; s__uncultured_Chloroflexi | CRT | 0 | 0 | 0 | 0 | 0.53944 |
| 65 | d__Bacteria; p__Chloroflexi; c__Dehalococcoidia; o__S085; f__S085; g__S085; s__uncultured_bacterium | CRT | 0 | 0 | 0 | 0 | 0.53339382 |
| 66 | d__Bacteria; p__Chloroflexi; c__Anaerolineae; o__RBG-13-54-9; f__RBG-13-54-9; g__RBG-13-54-9; s__uncultured_Chloroflexi | CRT | 0 | 0 | 0 | 0 | 0.412347173 |
| 67 | d__Bacteria; p__Chloroflexi; c__Dehalococcoidia; o__661239; f__661239; g__661239; s__uncultured_bacterium | CRT | 0 | 0 | 0.002329412 | 0 | 0.223281762 |
| 68 | d__Bacteria; p__Chloroflexi; c__Anaerolineae | CRT | 0 | 0 | 0 | 0 | 0.13502903 |
| 69 | d__Bacteria; p__Desulfobacterota; c__uncultured; o__uncultured; f__uncultured; g__uncultured | CRT | 0 | 0 | 0 | 0.005304767 | 0.148364636 |
| 70 | d__Bacteria; p__GAL15; c__GAL15; o__GAL15; f__GAL15; g__GAL15; s__uncultured_bacterium | CRT | 0 | 0 | 0 | 0.002930887 | 0.458231341 |
| 71 | d__Bacteria; p__GAL15; c__GAL15; o__GAL15; f__GAL15; g__GAL15; s__uncultured_bacterium | CRT | 0 | 0.00087266 | 0 | 0.012529595 | 0.267216282 |

**Table S3**. Continued.

| **No.** | **Taxonomy** | **Category** | **Mean relative abundance (%)** | | | | |
| --- | --- | --- | --- | --- | --- | --- | --- |
|  |  |  | **Mountain** | **Foothill** | **Basin­** | **Foothill-Mountain** | **Plain** |
| 72 | d__Bacteria; p__GAL15; c__GAL15; o__GAL15; f__GAL15; g__GAL15; s__uncultured_bacterium | CRT | 0 | 0 | 0 | 0.001769658 | 0.140746624 |
| 73 | d__Bacteria; p__Gemmatimonadota; c__Gemmatimonadetes; o__Gemmatimonadales; f__Gemmatimonadaceae; g__uncultured | CRT | 0 | 0 | 0.002937841 | 0.002652748 | 1.138884647 |
| 74 | d__Bacteria; p__Gemmatimonadota; c__Gemmatimonadetes; o__Gemmatimonadales; f__Gemmatimonadaceae; g__uncultured; s__uncultured_bacterium | CRT | 0 | 0 | 0 | 0 | 0.182361258 |
| 75 | d__Bacteria; p__Latescibacterota; c__Latescibacterota; o__Latescibacterota; f__Latescibacterota; g__Latescibacterota; s__uncultured_Latescibacteria | CRT | 0.002298121 | 0 | 0.009600689 | 0 | 0.771318927 |
| 76 | d__Bacteria; p__Latescibacterota; c__Latescibacteria; o__Latescibacterales; f__Latescibacteraceae; g__Latescibacteraceae; s__uncultured_bacterium | CRT | 0 | 0 | 0.001773135 | 0.014247936 | 0.647754255 |
| 77 | d__Bacteria; p__Latescibacterota; c__Latescibacterota; o__Latescibacterota; f__Latescibacterota; g__Latescibacterota; s__uncultured_soil | CRT | 0 | 0 | 0 | 0 | 0.516790848 |
| 78 | d__Bacteria; p__Latescibacterota; c__Latescibacterota; o__Latescibacterota; f__Latescibacterota; g__Latescibacterota; s__uncultured_soil | CRT | 0 | 0 | 0.006589246 | 0 | 0.423427908 |
| 79 | d__Bacteria; p__Latescibacterota; c__Latescibacterota; o__Latescibacterota; f__Latescibacterota; g__Latescibacterota; s__uncultured_soil | CRT | 0 | 0.001707077 | 0.002329412 | 0.031385073 | 0.346115207 |
| 80 | d__Bacteria; p__Latescibacterota; c__Latescibacterota; o__Latescibacterota; f__Latescibacterota; g__Latescibacterota | CRT | 0 | 0 | 0 | 0 | 0.296624936 |
| 81 | d__Bacteria; p__Latescibacterota; c__Latescibacterota; o__Latescibacterota; f__Latescibacterota; g__Latescibacterota | CRT | 0 | 0 | 0.009795665 | 0.01113459 | 0.213113426 |
| 82 | d__Bacteria; p__Latescibacterota; c__Latescibacterota; o__Latescibacterota; f__Latescibacterota; g__Latescibacterota | CRT | 0 | 0 | 0 | 0 | 0.200912905 |
| 83 | d__Bacteria; p__Latescibacterota; c__Latescibacterota; o__Latescibacterota; f__Latescibacterota; g__Latescibacterota; s__uncultured_soil | CRT | 0 | 0 | 0 | 0 | 0.150775508 |
| 84 | d__Bacteria; p__Methylomirabilota; c__Methylomirabilia; o__Rokubacteriales; f__Rokubacteriales; g__Rokubacteriales; s__uncultured_bacterium | CRT | 0 | 0 | 0.043948183 | 0.013649103 | 1.370635317 |
| 85 | d__Bacteria; p__Methylomirabilota; c__Methylomirabilia; o__Rokubacteriales; f__Rokubacteriales; g__Rokubacteriales; s__uncultured_bacterium | CRT | 0 | 0 | 0.00376878 | 0.002652748 | 0.710059387 |

**Table S3**. Continued.

| **No.** | **Taxonomy** | **Category** | **Mean relative abundance (%)** | | | | |
| --- | --- | --- | --- | --- | --- | --- | --- |
|  |  |  | **Mountain** | **Foothill** | **Basin­** | **Foothill-Mountain** | **Plain** |
| 86 | d__Bacteria; p__Methylomirabilota; c__Methylomirabilia; o__Rokubacteriales; f__WX65; g__WX65; s__uncultured_bacterium | CRT | 0 | 0 | 0.008154924 | 0.000883091 | 0.436222082 |
| 87 | d__Bacteria; p__Methylomirabilota; c__Methylomirabilia; o__Rokubacteriales; f__Rokubacteriales; g__Rokubacteriales; s__uncultured_bacterium | CRT | 0 | 0 | 0.001748797 | 0 | 0.318042211 |
| 88 | d__Bacteria; p__Myxococcota; c__Myxococcia; o__Myxococcales; f__Anaeromyxobacteraceae; g__Anaeromyxobacter; s__uncultured_bacterium | CRT | 0 | 0 | 0.002913503 | 0.005187288 | 0.674111691 |
| 89 | d__Bacteria; p__Myxococcota; c__bacteriap25; o__bacteriap25; f__bacteriap25; g__bacteriap25; s__uncultured_Syntrophobacterales | CRT | 0 | 0 | 0 | 0 | 0.379812622 |
| 90 | d__Bacteria; p__NB1-j; c__NB1-j; o__NB1-j; f__NB1-j; g__NB1-j | CRT | 0 | 0 | 0.0433958 | 0.013261864 | 1.004291391 |
| 91 | d__Bacteria; p__Nitrospirota | CRT | 0 | 0 | 0 | 0 | 1.412029248 |
| 92 | d__Bacteria; p__Nitrospirota; c__Leptospirillia; o__Leptospirillales; f__Leptospirillaceae; g__Leptospirillum; s__Nitrospirae_bacterium | CRT | 0 | 0 | 0.005232485 | 0.003793117 | 0.954505179 |
| 93 | d__Bacteria; p__Nitrospirota; c__Thermodesulfovibrionia; o__uncultured; f__uncultured; g__uncultured; s__uncultured_bacterium | CRT | 0 | 0 | 0 | 0 | 0.20889573 |
| 94 | d__Bacteria; p__Planctomycetota; c__Planctomycetes; o__Pirellulales; f__Pirellulaceae; g__uncultured | CRT | 0 | 0 | 0 | 0 | 0.997987132 |
| 95 | d__Bacteria; p__Planctomycetota; c__Planctomycetes; o__Pirellulales; f__Pirellulaceae; g__uncultured | CRT | 0 | 0 | 0.001164706 | 0.014145998 | 0.941579967 |
| 96 | d__Bacteria; p__Planctomycetota; c__Planctomycetes; o__Pirellulales; f__Pirellulaceae; g__Pirellula; s__uncultured_bacterium | CRT | 0 | 0 | 0 | 0.001140369 | 0.833374847 |
| 97 | d__Bacteria; p__Planctomycetota; c__Planctomycetes; o__Pirellulales; f__Pirellulaceae | CRT | 0 | 0 | 0 | 0 | 0.770553072 |
| 98 | d__Bacteria; p__Planctomycetota; c__Planctomycetes; o__Pirellulales; f__Pirellulaceae; g__Pirellula | CRT | 0 | 0 | 0 | 0 | 0.659023985 |
| 99 | d__Bacteria; p__Planctomycetota; c__Planctomycetes; o__Planctomycetales; f__uncultured; g__uncultured; s__uncultured_bacterium | CRT | 0 | 0.006885707 | 0.010510273 | 0.004919962 | 0.628147211 |
| 100 | d__Bacteria; p__Planctomycetota; c__Planctomycetes; o__Pirellulales; f__Pirellulaceae; g__Rhodopirellula | CRT | 0 | 0 | 0 | 0 | 0.583175129 |

**Table S3.** Continued.

| **No.** | **Taxonomy** | **Category** | **Mean relative abundance (%)** | | | | |
| --- | --- | --- | --- | --- | --- | --- | --- |
|  |  |  | **Mountain** | **Foothill** | **Basin­** | **Foothill-Mountain** | **Plain** |
| 101 | d__Bacteria; p__Planctomycetota; c__Planctomycetes; o__Pirellulales; f__Pirellulaceae; g__Pirellula | CRT | 0 | 0 | 0 | 0.002652748 | 0.509202388 |
| 102 | d__Bacteria; p__Planctomycetota; c__Planctomycetes; o__Pirellulales; f__Pirellulaceae; g__Rhodopirellula | CRT | 0 | 0 | 0 | 0.011493632 | 0.474637599 |
| 103 | d__Bacteria; p__Planctomycetota; c__Planctomycetes; o__Pirellulales; f__Pirellulaceae; g__Blastopirellula; s__bacterium_enrichment | CRT | 0 | 0 | 0 | 0 | 0.461514908 |
| 104 | d__Bacteria; p__Planctomycetota; c__Planctomycetes; o__Planctomycetales; f__uncultured; g__uncultured; s__uncultured_bacterium | CRT | 0 | 0 | 0 | 0.007514927 | 0.440625992 |
| 105 | d__Bacteria; p__Planctomycetota; c__Planctomycetes; o__Pirellulales; f__Pirellulaceae; g__uncultured | CRT | 0 | 0.00087266 | 0 | 0.007800019 | 0.435303459 |
| 106 | d__Bacteria; p__Planctomycetota; c__OM190; o__OM190; f__OM190; g__OM190; s__uncultured_bacterium | CRT | 0 | 0 | 0 | 0 | 0.411425247 |
| 107 | d__Bacteria; p__Planctomycetota; c__OM190; o__OM190; f__OM190; g__OM190; s__uncultured_bacterium | CRT | 0 | 0 | 0 | 0 | 0.326151485 |
| 108 | d__Bacteria; p__Planctomycetota; c__vadinHA49; o__vadinHA49; f__vadinHA49; g__vadinHA49; s__uncultured_bacterium | CRT | 0 | 0 | 0 | 0 | 0.300152987 |
| 109 | d__Bacteria; p__Planctomycetota; c__Planctomycetes; o__Pirellulales; f__Pirellulaceae; g__uncultured | CRT | 0 | 0 | 0.00953331 | 0.004930009 | 0.268154896 |
| 110 | d__Bacteria; p__Planctomycetota; c__Planctomycetes; o__Pirellulales; f__Pirellulaceae; g__Blastopirellula; s__uncultured_bacterium | CRT | 0 | 0 | 0 | 0 | 0.22637922 |
| 111 | d__Bacteria; p__Planctomycetota; c__OM190; o__OM190; f__OM190; g__OM190; s__uncultured_bacterium | CRT | 0 | 0 | 0.001773135 | 0.001366357 | 0.210362147 |
| 112 | d__Bacteria; p__Planctomycetota; c__Planctomycetes; o__Pirellulales; f__Pirellulaceae; g__Blastopirellula; s__uncultured_bacterium | CRT | 0 | 0 | 0 | 0 | 0.209302508 |
| 113 | d__Bacteria; p__Planctomycetota; c__Planctomycetes; o__Pirellulales; f__Pirellulaceae; g__Blastopirellula; s__uncultured_bacterium | CRT | 0 | 0 | 0.001960878 | 0 | 0.198910931 |
| 114 | d__Bacteria; p__Planctomycetota; c__Pla3_lineage; o__Pla3_lineage; f__Pla3_lineage; g__Pla3_lineage | CRT | 0 | 0 | 0 | 0 | 0.188328829 |

**Table S3**. Continued.

| **No.** | **Taxonomy** | **Category** | **Mean relative abundance (%)** | | | | |
| --- | --- | --- | --- | --- | --- | --- | --- |
|  |  |  | **Mountain** | **Foothill** | **Basin­** | **Foothill-Mountain** | **Plain** |
| 115 | d__Bacteria; p__Planctomycetota; c__Phycisphaerae; o__Phycisphaerales; f__Phycisphaeraceae; g__Urania-1B-19_marine_sediment_group | CRT | 0 | 0 | 0 | 0 | 0.175762069 |
| 116 | d__Bacteria; p__Proteobacteria; c__Gammaproteobacteria; o__Burkholderiales; f__TRA3-20; g__TRA3-20; s__uncultured_Nitrosomonadaceae | CRAT | 0 | 0 | 0.002937841 | 0 | 4.546421582 |
| 117 | d__Bacteria; p__Proteobacteria; c__Gammaproteobacteria; o__Burkholderiales; f__Nitrosomonadaceae; g__MND1 | CRAT | 0 | 0 | 0.074211453 | 0.210714584 | 4.122896708 |
| 118 | d__Bacteria; p__Proteobacteria; c__Gammaproteobacteria; o__PLTA13; f__PLTA13; g__PLTA13 | CRAT | 0 | 0 | 0.001164706 | 0 | 2.976204498 |
| 119 | d__Bacteria; p__Proteobacteria; c__Gammaproteobacteria; o__Gammaproteobacteria_Incertae_Sedis; f__Unknown_Family; g__Acidibacter | CRT | 0 | 0 | 0.05964411 | 0.263061167 | 2.075681183 |
| 120 | d__Bacteria; p__Proteobacteria; c__Gammaproteobacteria; o__Burkholderiales; f__Nitrosomonadaceae; g__Ellin6067 | CRT | 0 | 0 | 0.001960878 | 0.037368637 | 1.42209429 |
| 121 | d__Bacteria; p__Proteobacteria; c__Gammaproteobacteria; o__KF-JG30-C25; f__KF-JG30-C25; g__KF-JG30-C25; s__uncultured_organism | CRT | 0 | 0 | 0.0050203 | 0.037963437 | 1.077522439 |
| 122 | d__Bacteria; p__Proteobacteria; c__Gammaproteobacteria; o__Burkholderiales; f__Comamonadaceae | CRT | 0 | 0 | 0.020969785 | 0.002652748 | 0.869153017 |
| 123 | d__Bacteria; p__Proteobacteria; c__Gammaproteobacteria; o__KF-JG30-C25; f__KF-JG30-C25; g__KF-JG30-C25; s__uncultured_organism | CRT | 0 | 0 | 0.000584091 | 0.006445136 | 0.871203804 |
| 124 | d__Bacteria; p__Proteobacteria; c__Gammaproteobacteria; o__Burkholderiales; f__Nitrosomonadaceae; g__GOUTA6 | CRT | 0 | 0 | 0.02067607 | 0 | 0.828718248 |
| 125 | d__Bacteria; p__Proteobacteria; c__Gammaproteobacteria; o__Burkholderiales; f__Nitrosomonadaceae; g__MND1 | CRT | 0 | 0 | 0.001960878 | 0 | 0.741548171 |
| 126 | d__Bacteria; p__Proteobacteria; c__Gammaproteobacteria | CRT | 0 | 0 | 0 | 0.045190177 | 0.605494373 |
| 127 | d__Bacteria; p__Proteobacteria; c__Gammaproteobacteria; o__CCD24; f__CCD24; g__CCD24 | CRT | 0 | 0 | 0 | 0 | 0.613330759 |
| 128 | d__Bacteria; p__Proteobacteria; c__Alphaproteobacteria; o__Rhizobiales; f__Hyphomicrobiaceae | CRT | 0 | 0.002305075 | 0.001748797 | 0.022612159 | 0.582191909 |

**Table S3**. Continued.

| **No.** | **Taxonomy** | **Category** | **Mean relative abundance (%)** | | | | |
| --- | --- | --- | --- | --- | --- | --- | --- |
|  |  |  | **Mountain** | **Foothill** | **Basin­** | **Foothill-Mountain** | **Plain** |
| 130 | d__Bacteria; p__Proteobacteria; c__Gammaproteobacteria; o__JG36-GS-52; f__JG36-GS-52; g__JG36-GS-52 | CRT | 0 | 0 | 0.000980439 | 0.020116594 | 0.399431799 |
| 131 | d__Bacteria; p__Proteobacteria; c__Alphaproteobacteria; o__Ferrovibrionales; f__Ferrovibrionales; g__uncultured; s__uncultured_bacterium | CRT | 0 | 0 | 0.005242464 | 0.015298188 | 0.286585413 |
| 132 | d__Bacteria; p__Proteobacteria; c__Gammaproteobacteria; o__Burkholderiales; f__Nitrosomonadaceae; g__GOUTA6; s__uncultured_bacterium | CRT | 0 | 0 | 0.000584091 | 0.002847446 | 0.26965083 |
| 133 | d__Bacteria; p__Schekmanbacteria; c__Schekmanbacteria; o__Schekmanbacteria; f__Schekmanbacteria; g__Schekmanbacteria; s__uncultured_bacterium | CRT | 0 | 0 | 0.002604074 | 0.006553192 | 0.295733014 |
| 134 | d__Bacteria; p__Zixibacteria; c__Zixibacteria; o__Zixibacteria; f__Zixibacteria; g__Zixibacteria; s__candidate_division | CRT | 0 | 0 | 0 | 0 | 0.523042434 |
| 135 | d__Bacteria; p__Zixibacteria; c__Zixibacteria; o__Zixibacteria; f__Zixibacteria; g__Zixibacteria; s__uncultured_bacterium | CRT | 0 | 0 | 0 | 0.002277261 | 0.490076559 |
| 136 | d__Bacteria; p__Zixibacteria; c__Zixibacteria; o__Zixibacteria; f__Zixibacteria; g__Zixibacteria; s__uncultured_bacterium | CRT | 0 | 0 | 0 | 0 | 0.376407404 |

**Table S4**. Incidence of strong and significant intra- and inter-phylum co-occurrence patterns. The observed incidence (O) of co-occurrence between phyla was calculated as the number of observed edges divided by total number of edges in the realistic networks, while the random incidence (R) was calculated by considering the node number of each phylum, and the random association among them. Only those observed co-occurrence patterns with O > 0.5% and O/R ratio > 1 are shown.

| **Phylum-1** | **Phylum-2** | **Phylum-1 node number** | **Phylum-2 node number** | **Edge number** | **O (%)** | **R (%)** | **O/R ratio** |
| --- | --- | --- | --- | --- | --- | --- | --- |
| Planctomycetota | Planctomycetota | 131 | 131 | 695 | 3.364802711 | 1.160293569 | 2.899958082 |
| Planctomycetota | Zixibacteria | 131 | 18 | 173 | 0.837569596 | 0.321312065 | 2.606716917 |
| Acidobacteriota | GAL15 | 251 | 16 | 286 | 1.384652626 | 0.547238869 | 2.5302527 |
| Acidobacteriota | Zixibacteria | 251 | 18 | 304 | 1.471798596 | 0.615643728 | 2.39066611 |
| Acidobacteriota | Planctomycetota | 251 | 131 | 1751 | 8.477366255 | 4.480518242 | 1.892050383 |
| Latescibacterota | Planctomycetota | 52 | 131 | 335 | 1.621883321 | 0.928234855 | 1.747276902 |
| Acidobacteriota | Methylomirabilota | 251 | 20 | 243 | 1.176470588 | 0.684048587 | 1.719864073 |
| Acidobacteriota | Acidobacteriota | 251 | 251 | 1472 | 7.126603728 | 4.275303666 | 1.666923401 |
| Acidobacteriota | Latescibacterota | 251 | 52 | 548 | 2.653110627 | 1.778526325 | 1.491746616 |
| Acidobacteriota | Gemmatimonadota | 251 | 24 | 250 | 1.210360687 | 0.820858304 | 1.474506236 |
| Proteobacteria | Zixibacteria | 224 | 18 | 167 | 0.808520939 | 0.549419104 | 1.47159233 |
| Actinobacteriota | Planctomycetota | 42 | 131 | 201 | 0.973129993 | 0.749728152 | 1.297977127 |
| Acidobacteriota | Bacteroidota | 251 | 129 | 1106 | 5.354635681 | 4.412113383 | 1.213621504 |
| Bacteroidota | Planctomycetota | 129 | 131 | 576 | 2.788671024 | 2.302736467 | 1.211024824 |
| Bacteroidota | Bacteroidota | 129 | 129 | 281 | 1.360445413 | 1.125001022 | 1.209283713 |
| Bacteroidota | Latescibacterota | 129 | 52 | 217 | 1.050593077 | 0.91406333 | 1.149365741 |
| Acidobacteriota | Actinobacteriota | 251 | 42 | 339 | 1.641249092 | 1.436502032 | 1.142531689 |
| Acidobacteriota | Proteobacteria | 251 | 224 | 1592 | 7.707576858 | 7.661344169 | 1.00603454 |

**Table S5**. Partial Mantel analysis of βNTI and environmental factors with geographic distance as the control variable.

| Environmental factors | ART (Mantel’s *r*) | |  | CRT (Mantel’s *r*) | |  | CRAT (Mantel’s *r*) | |
| --- | --- | --- | --- | --- | --- | --- | --- | --- |
|  | Spring | Autumn |  | Spring | Autumn |  | Spring | Autumn |
| WT | 0.076 | 0.025 |  | 0.022 | -0.041 |  | -0.019 | 0.026 |
| pH | 0.085 | 0.174 |  | 0.184 | **0.193*** |  | 0.048 | 0.004 |
| NH_3_-N | **0.193*** | 0.138 |  | 0.108 | 0.123 |  | -0.121 | -0.019 |
| NO_3_-N | -0.018 | -0.104 |  | 0.019 | -0.045 |  | -0.018 | 0.107 |
| TN | 0.126 | 0.085 |  | 0.036 | 0.099 |  | -0.074 | -0.060 |
| TOC | **0.135*** | 0.121 |  | **0.169**** | **0.131*** |  | -0.030 | -0.137 |
| TP | 0.019 | 0.102 |  | 0.106 | -0.029 |  | 0.023 | 0.001 |
| River flow | 0.055 | 0.022 |  | -0.033 | 0.105 |  | 0.030 | -0.069 |
| Channel slope | 0.036 | 0.067 |  | **0.107*** | -0.141 |  | -0.001 | -0.107 |
